# Modulated self-assembly of three flexible Cr(iii) PCPs for SO_2_ adsorption and detection†

**DOI:** 10.1039/d3cc01685d

**Published:** 2023-06-01

**Authors:** Valeria B. López-Cervantes, Dominic Bara, Ana Yañez-Aulestia, Eva Martínez-Ahumada, Alfredo López-Olvera, Yoarhy A. Amador-Sánchez, Diego Solis-Ibarra, Elí Sánchez-González, Ilich A. Ibarra, Ross S. Forgan

**Affiliations:** a Laboratorio de Fisicoquímica y Reactividad de Superficies (LaFReS), Instituto de Investigaciones en Materiales, Universidad Nacional Autónoma de México, Circuito Exterior s/n, CU, Del. Coyoacan 04510 Ciudad de Mexico Mexico argel@unam.mx +52-55-5622-4595; b WestCHEM School of Chemistry, University of Glasgow Glasgow G12 8QQ UK ross.forgan@glasgow.ac.uk

## Abstract

Modulated self-assembly protocols are used to develop facile, HF-free syntheses of the archetypal flexible PCP, MIL-53(Cr), and novel isoreticular analogues MIL-53(Cr)-Br and MIL-53(Cr)-NO_2_. All three PCPs show good SO_2_ uptake (298 K, 1 bar) and high chemical stabilities against dry and wet SO_2_. Solid-state photoluminescence spectroscopy indicates all three PCPs exhibit turn-off sensing of SO_2_, in particular MIL-53(Cr)-Br, which shows a 2.7-fold decrease in emission on exposure to SO_2_ at room temperature, indicating potential sensing applications.

## Abstract 2

Modulated self-assembly protocols are used to develop facile, HF-free syntheses of the archetypal flexible PCP, MIL-53(Cr), and novel isoreticular analogues MIL-53(Cr)-Br and MIL-53(Cr)-NO_2_, which adsorb and sense SO_2_.

## Graphical abstract

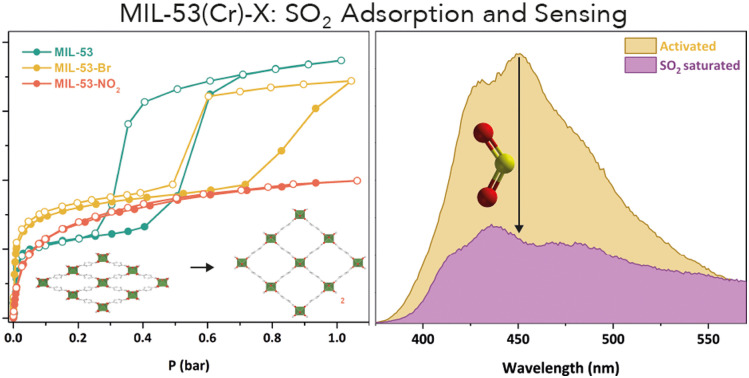

Porous coordination polymers (PCPs), a class of hybrid materials comprised of extended metal–ligand coordination networks, have attracted attention due to their high porosity and tuneable and adaptable structures.^1^ Many “flexible” PCPs undergo reversible crystal-to-crystal structural transformations upon guest inclusion and removal,^2^ combining a highly ordered network with structural transformability^2*a*^ and/or local molecular motion, often with relatively large movements of >5–10 Å.^3^ Phase changes can occur in response to external stimuli, *e.g.*, light, temperature, pressure, electric and/or magnetic fields and guest adsorption/desorption.^4^ The most common dynamic mode is “breathing”,^2*b*,4*c*^ often characterised by a phase transition between two or more distinct states causing a unit cell volume change.^3^ “Gate-opening”^4*c*^ generally results in the pore opening in the presence of adsorbate molecules, offering the attractive possibility of pressure-swing processes for selective gas adsorption and separation.^5^

The MIL-53 isoreticular series comprises one of the most investigated families of flexible PCPs.^6^ One-dimensional chains of octahedral CrO_4_(OH)_2_ units containing axially bridging μ_2_-OH moieties and four equatorial carboxylate oxygen atoms from four benzene-1,4-dicarboxylate (BDC) ligands are connected into a diamondoid net with **sra** topology. First isolated as the Cr congener,^6*a*,6*b*^ examples with Sc, V, Fe, Al, In, and Ga, as well as derivatives with functionalised BDC ligands, have since been synthesised.^6*c*^ Introduction of –Br and –NO_2_ substituents to MIL-53(Al) and MIL-53(In) tuned their flexibilities and gas uptakes; locating functional groups within the diamondoid channel stiffens the pore due to steric hindrance.^7^ There are relatively few reports, however, of functionalised MIL-53(Cr) derivatives.^8^

The functional diversity and structural adjustability of PCPs mean they can achieve efficient and specific host–guest recognition, while the presence of many π and n electrons can induce fluorescence, making PCPs attractive candidates as sensors.^9^ It has been suggested that flexible PCPs could be more suited to luminescent detection compared to rigid materials,^10^ for example, [Zn_2_(BDC)_2_(dpNDI)] has a doubly-interpenetrated dynamic structure and exhibits strong fluorescence only if there is movement between its two nets; if it remains rigid, no luminescence is observed.^11^ However, research on flexible PCPs that can be used as luminescent sensors is limited. Herein we report the modulated self-assembly of three PCPs of the flexible MIL-53(Cr) family, avoiding the use of HF in their syntheses, and detail the SO_2_ uptake and detection behaviours of these PCPs.

MIL-53(Cr) and its novel –Br and –NO_2_ substituted analogues were obtained by a modification of the original literature protocol (ESI,† Section S2),^6*a*^ where we applied the principles of modulated self-assembly^12^ that were previously successful for the synthesis of MIL-53(Fe).^13^ Equimolar amounts of the linker and CrCl_3_·6H_2_O, with concentrated HCl as modulator, were reacted hydrothermally at 220 °C in a Teflon-lined autoclave for 72 h to yield as-synthesised (as) PCPs after washing. Samples were heated in DMF for 16 h at 220 °C to isolate the PCPs as DMF solvates (DMF),^14^ removing any residual bound linker, and then refluxed in MeOH for 16 h before drying under vacuum (MeOH). Samples were activated (act) for adsorption experiments by degassing on a turbomolecular vacuum pump at 150 °C for 20 h. Comparison of powder X-ray diffractograms (Fig. 1 and ESI,† Section S3) showed all three DMF solvates were structurally similar to the MIL-53(Fe) DMF solvate reported previously,^13^ while washing with MeOH allowed the samples to close. MIL-53(Cr) was isolated as its hydrated narrow pore form, MIL-53_lt (Fig. 1a).^6*a*,6*b*^ Comparison with the predicted diffractograms from the crystal structures of the In analogues^7*b*^ confirmed the formation of MIL-53(Cr)-Br (Fig. 1b) and MIL-53(Cr)-NO_2_ (Fig. 1c). However, upon drying, they were isolated in a more open form than that of both the Al and In analogues,^7^ indicating that the nature of the metal ion influences the behaviour of these two new PCPs.

**Fig. 1:**
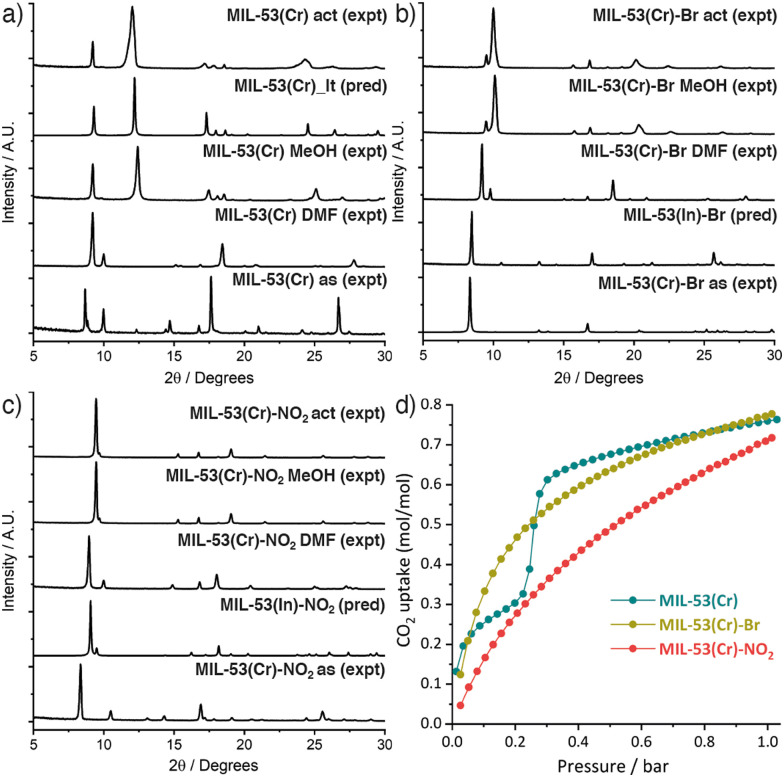
Stacked powder X-ray diffractograms of (a) MIL-53(Cr), (b) MIL-53(Cr)-Br, and (c) MIL-53(Cr)-NO_2_ across their respective synthesis and activation procedures (as = as-synthesised, act = activated). (d) CO_2_ adsorption isotherms (273 K) of the three MOFs.

Only MIL-53(Cr) adsorbed N_2_ at 77 K (BET area = 1411 m^2^ g^−1^) while MIL-53(Cr)-Br and MIL-53(Cr)-NO_2_ again behaved differently from the Al and In analogues in adsorbing negligible amounts of N_2_. In contrast, all three Cr MOFs showed good CO_2_ uptake at 273 K (Fig. 1d) and 298 K (Fig. S20, ESI†) confirming their porosity, with MIL-53(Cr) exhibiting a characteristic stepped adsorption isotherm consistent with a structural transition to an open pore phase.^15^ The CO_2_ uptakes of the functionalised MOFs are comparable to the Al analogues,^7*a*^ as are their isosteric enthalpies of CO_2_ adsorption (∼20–40 kJ mol^−1^, Fig. S22, ESI†). Using HCl as a modulator in hydrothermal syntheses avoids the highly toxic HF, which has only been achieved previously through mechanochemical approaches,^16^ and is clearly applicable to producing a range of MIL-53(Cr) derivatives.

Adsorption–desorption SO_2_ isotherms (298 K, 0–1 bar) were collected on samples that had been further activated at 473 K under vacuum for 2 h (Fig. 2). The SO_2_ adsorption-desorption isotherm for MIL-53(Cr) again shows a stepwise profile, indicative of guest-induced structural changes in the material. After a sharp initial SO_2_ uptake at low pressure (*ca.* 0.66 mol mol^−1^), gate-opening is observed at around 0.4 bar, with SO_2_ uptake reaching 1.86 mol mol^−1^ at 0.6 bar and gradually increasing to reach the total SO_2_ capture value of 1.87 mol mol^−1^ at 1 bar. Significant hysteresis is observed on the desorption branch, with the onset of desorption and concomitant gate-closing at less than 0.4 bar.

**Fig. 2:**
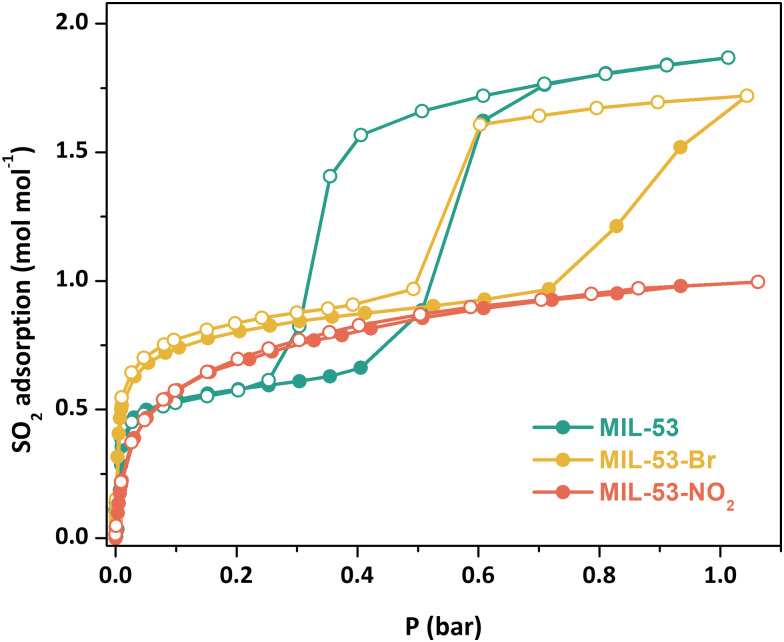
Experimental SO_2_ adsorption–desorption isotherms for fully activated MIL-53(Cr) (green), MIL-53(Cr)-Br (yellow), and MIL-53(Cr)-NO_2_ (red) samples at 298 K and up to 1 bar. Filled circles = adsorption; open circles = desorption.

MIL-53(Cr)-Br shows a similar stepwise profile, with a sharp increase in SO_2_ adsorption at low pressure reaching 0.97 mol mol^−1^ at 0.72 bar, where gate-opening occurs, with a second increase in SO_2_ capture reaching a total uptake of 1.72 mol mol^−1^ at 1 bar. Hysteresis in the desorption branch is again present, with the onset of gate-closing occurring around 0.6 bar. MIL-53(Cr)-Br requires a higher SO_2_ pressure to achieve the gate opening effect than the unsubstituted MOF, likely due to the steric bulk of the Br functional group modifying the inclination of the phenyl ring, making ligand rotation more difficult.^7,17^ It is also notable that the initial SO_2_ uptake for MIL-53(Cr)-Br is higher than MIL-53(Cr), likely due to it being held in a more open pore state because of the Br group, whilst at 1 bar SO_2_ uptake is slightly lower than MIL-53(Cr), as would be expected given the latter would have a larger pore volume.

In contrast, MIL-53(Cr)-NO_2_ shows a typical type-I adsorption-desorption isotherm, reaching a total SO_2_ uptake of 0.99 mol mol^−1^ at 1 bar. The NO_2_ groups are larger than the Br groups, making ligand rotation even more difficult due to the higher steric hindrance, thus the gating phenomenon presumably occurs at higher pressure.^17*a*^ The flexibilities of functionalised MIL-53(Al) derivatives also depend on the chemical nature of the functional groups,^17*b*^ in particular hydrogen bonding between the functional groups and the OH ligands; strong interactions between the NO_2_ group and the μ_2_-OH may hinder the easy opening of the pore.

Powder X-ray diffractograms of the three materials collected after SO_2_ adsorption and desorption (ESI,† Section S7) revealed that they were stable to SO_2_ exposure, and the three PCPs returned to their pre-activated structures. Similarly, infrared spectra of the PCPs showed no changes after SO_2_ exposure.

In our previous examination of SO_2_ adsorption by MIL-53(Al), Monte Carlo simulations showed that strong interactions exist between SO_2_ molecules *via* their O-atoms, and the H-atom of the μ_2_-OH group in the inorganic secondary building unit; there are also interactions between SO_2_ and the organic linker.^18^ Thus, we propose a similar SO_2_ adsorption mechanism for the MIL-53(Cr) material in this work. In addition to this binding motif, electronegative substituents on PCPs, such as fluoro units, have been observed to enhance SO_2_ uptake through electrostatic attraction between S^*δ*+^ and F^*δ*−^ units in DFT calculations.^19^ Thus, we propose a similar SO_2_ adsorption mechanism for the substituted PCPs, in which the main adsorption interaction is between the S^*δ*+^ of SO_2_ and Br^*δ*−^ for MIL-53(Cr)-Br, and O^*δ*−^ atoms of the highly polar –NO_2_ groups^20^ of MIL-53(Cr)-NO_2_, respectively.

We also evaluated the SO_2_ selectivity over CO_2_ for these materials by comparison of single component adsorption isotherms (298 K, 1 bar, Fig. S31, ESI†). For all three samples, the SO_2_ adsorbed amount is much higher than the CO_2_ over the whole pressure range. The pyIAST python package^21^ was used to apply the ideal adsorbed solution theory (IAST) model to the single component adsorption isotherms to calculate the SO_2_/CO_2_ selectivities at 1 bar for different molar SO_2_/CO_2_ compositions. These results are summarized in Table S1 (ESI†).

The SO_2_ uptake of MIL-53(Cr) and its derivatives compare reasonably well to other systems, but are lower than MIL-53(Al) and some larger pore MOFs.^18,19^ Thus, we sought to explore the possibility of using these materials as efficient fluorescent SO_2_ detectors. Under UV light irradiation at *λ*_ex_ = 300 nm (chosen based on solid-state UV-vis spectroscopy experiments, Fig. S15, ESI†), MIL-53(Cr) displays a broad photoluminescence peak centred at *λ*_max_ = 415 nm (Fig. 3a), whose origin can be attributed to the organic linker.^9^ The emission of MIL-53(Cr) is similar to that of the free ligand (Fig. S35 (ESI†), *λ*_max_ = 383 nm) with a slight red shift, indicating metal-to-ligand charge transfer that perturbs the ligand energy levels.^9^ After exposure to SO_2_ in our homemade *in situ* adsorption system, the MIL-53(Cr) sample was packed in a quartz holder for solids to measure its photoluminescence properties (ESI,† Section S11), and a shift in emission to *λ*_max_ = 420 nm was observed, with emission intensity decreasing 1.4-fold (quenching). For MIL-53(Cr)-Br (*λ*_ex_ = 360 nm), the maximum of the photoluminescence for the activated sample is located at *λ*_max_ = 450 nm and changes to *λ*_max_ = 436 nm after the SO_2_ exposure (Fig. 3b). Remarkably, the emission intensity is quenched 2.7-fold, suggesting potential application as a turn-off sensor. Finally, MIL-53(Cr)-NO_2_ (*λ*_ex_ = 350 nm) exhibits broad photoluminescence centred at *λ*_max_ = 507 nm and, although small, a 1.2-fold decrease in intensity is also observed upon SO_2_ exposure (Fig. 3c). The quenching observed in all three materials can be attributed to the electronic effect that SO_2_ exerts on their structures after exposure.^9,22^ Further, the fact that the most significant quenching effect is observed for MIL-53(Cr)-Br is consistent with previous observations of halogenated PCPs showing stronger affinities for SO_2_.^19^ When these three materials were exposed to H_2_O and CO_2_ (Fig. S36, ESI†), or left for some time after activation (Fig. S37, ESI†) no significant changes in the shape or intensity of the emission were observed with respect to the spectrum of the activated samples. These facts support that the change in fluorescence is due to the adsorption of SO_2_ and not to other factors, and that there is some selectivity in the response.

**Fig. 3:**
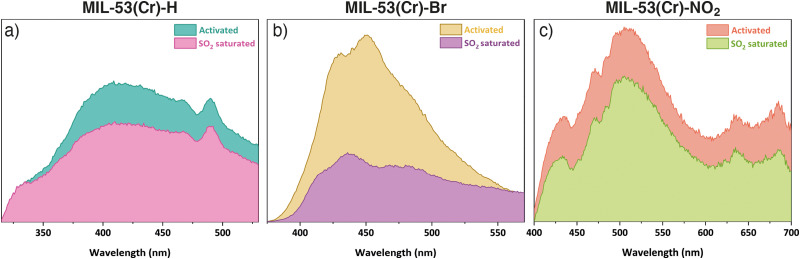
Solid-state emission spectra of activated and SO_2_ saturated samples of (a) MIL-53(Cr), (b) MIL-53(Cr)-Br, and (c) MIL-53(Cr)-NO_2_.

In this work, HCl-modulated hydrothermal syntheses of MIL-53(Cr), MIL-53(Cr)-Br and MIL-53(Cr)-NO_2_ were developed, which are comparable to existing green mechanochemical routes to MIL-53(Cr)^16^ but with enhanced substrate scope offering a new route into this valuable isoreticular series. All three materials exhibit reversible SO_2_ adsorption–desorption with high chemical stability to dry and wet SO_2_. Changes in the emission spectra of the MOFs after the SO_2_ adsorption, particularly the large quenching observed for MIL-53(Cr)-Br, suggest this material has potential as a sensor for SO_2_.

This project received financial support in part from the European Research Council (ERC) under the European Union's Horizon 2020 Programme for Research and Innovation (grant agreement no. 677289, SCoTMOF, ERC-2015-STG). I.A.I. thanks PAPIIT UNAM (IN201123), México, for financial support. We thank Mohammad Reza Alizadeh Kiapi (University of Cambridge) for assistance with BETSI analysis^23^ of gas adsorption data, and Matthew Liddle (University of Glasgow) for thermogravimetric analysis. The data which underpin this submission are available at http://dx.doi.org/10.5525/gla.researchdata.1446.

## Conflicts of interest

There are no conflicts to declare.

## Supplementary Material

CC-059-D3CC01685D-s001
