# Supplementary material for: Modulated self-assembly of three flexible Cr(iii) PCPs for SO2 adsorption and detection
Source: Chem Commun (Camb). 2023 Jun 1;59(52):8115–8. doi: 10.1039/d3cc01685d (PMC10297829; doi:10.1039/d3cc01685d)
Supplement: CC-059-D3CC01685D-s001 [file CC-059-D3CC01685D-s001.pdf]

Electronic Supplementary Material (ESI) for ChemComm.
This journal is © The Royal Society of Chemistry 2023

Electronic Supplementary Information

Modulated Self-Assembly of Three Flexible Cr(III) PCPs for SO_2 Adsorption and Detection

Valeria B. López-Cervantes,‡[a] Dominic Bara,‡[b] Ana Yañez-Aulestia,‡[a] Eva Martínez-Ahumada,[a] Alfredo López-Olvera,[a] Yoarhy A. Amador-Sánchez,[a] Diego Solis-Ibarra,*[a] Elí Sánchez-González,[a] Ilich A. Ibarra*[a] and Ross S. Forgan*[b]

[a] Laboratorio de Fisicoquímica y Reactividad de Superficies (LaFReS), Instituto de Investigaciones en Materiales, Universidad Nacional Autónoma de México, Circuito Exterior s/n, CU, Del. Coyoacan, 04510, Ciudad de México, México. E-mail: argel@unam.mx and diego.solis@unam.mx Fax: +52-55-5622-4595

[b] WestCHEM School of Chemistry, University of Glasgow, Glasgow, UK. E-mail: ross.forgan@glasgow.ac.uk

‡ These authors contributed equally to this work.

* Corresponding Authors

Table of Contents

S1. Materials and Methods.. S3

S2. Synthesis.. S4

S3. Powder X-Ray Diffraction Analyses.. S5

S4. Thermogravimetric Analysis.. S21

S5. Adsorption Isotherms.. S22

S6. Custom *ex situ* SO_2 Adsorption System.. S29

S7. Characterisation after SO_2 Adsorption.. S30

S8. Comparison of SO_2 and CO_2 Isotherms.. S36

S9. Ideal Adsorbed Solution Theory (IAST).. S37

S10. UV-vis Spectroscopy... S39

S11. Fluorescence Spectroscopy... S40

S12. References.. S43

S1. Materials and Methods

Chemicals and solvents were purchased from Alfa Aesar, Fluorochem, Tokyo Chemical Industry, Sigma-Aldrich, Strem and VWR and used without further purification. Samples were synthesised and characterised at the University of Glasgow, prior to SO_2 adsorption experiments and further characterisation at Universidad Nacional Autónoma de México.

Powder X-Ray Diffraction (PXRD): PXRD measurements carried out at the University of Glasgow at 298 K used a Rigaku Miniflex diffractometer (λ (CuKα(mean)) = 1.54183 Å) on a zero-background sample plate on a rotating sample stage. Data were collected from 3–45° at a step size of 0.01°. Pawley fitting and indexing were carried out using GSAS-II.[S1]

Thermogravimetric Analysis (TGA): TGA measurements were carried out at the University of Glasgow using a TA Instruments TGA 5500 thermal analyser. Measurements were carried out in air.

Gas Uptake: N_2 adsorption and desorption isotherms up to 1 bar were carried out at 77 K on a Quantachrome Autosorb iQ gas sorption analyser at the University of Glasgow. CO_2 adsorption and desorption isotherms up to 1 bar were carried out at 273 K and 298 K on a Quantachrome Autosorb iQ gas sorption analyser at the University of Glasgow. Samples were degassed under vacuum at 150 °C for 20 h using the internal turbo pump. BET surface areas were calculated from the isotherms using BETSI software package.[S2]

SO_2 Uptake: SO_2 adsorption experiments were carried out using a Dynamic Gravimetric Gas/Vapour Sorption Analyser, DVS vacuum (Surface Measurement Systems Ltd) at Universidad Nacional Autónoma de México. MIL-53(Cr), MIL-53(Cr)-Br, and MIL-53(Cr)-NO_2 samples were degassed in vacuum (1×10^{-6} Torr) at 473 K for 2 h. After cooling to 25°C, the isotherms were measured from 0.0007 to 100% P/P0 of SO_2 gas, followed by the desorption isotherm.

PXRD After SO_2 Adsorption: PRXD patterns after SO_2 exposure were recorded at Universidad Nacional Autónoma de México on a Siemes Diffractometer, D5000 with a Cu-Kα1 radiation (λ = 1.5406 Å) using a nickel filter. Patterns were recorded in the 5–50° 2θ range with a step scan of 0.02° and a scan rate of 0.08° min^{-1}.

IR Spectroscopy: FT-IR spectra were obtained in the range of 4000-400 cm^{-1} on a Bruker Tensor 27 spectrometer with a Golden Gate Single Reflection diamond ATR cell at Universidad Nacional Autónoma de México.

UV-vis Spectroscopy: Absorption measurements were recorded at Universidad Nacional Autónoma de México using a Shimadzu spectrophotometer UV-2600 equipped with an ISR-2600Plus integrating sphere and a $BaSO_4$ blank.

Fluorescence: The fluorescence experiments were carried out in an Edinburgh Instruments FS5 Spectrofluorometer at Universidad Nacional Autónoma de México, coupled with the SC-10 solid-state sample holder. The samples were packed into quartz sample holders and positioned into the instrument. Both the activated and saturated samples were packed right after being taken out of the activation and saturation processes.

S2. Synthesis

MIL-53(Cr): Terephthalic acid (1.66 g, 1 mmol) and chromium (III) chloride hexahydrate (2.66 g, 1 mmol) were added to a 200 mL Teflon autoclave liner and water (100 mL) was added along with 12 M HCl (1.5 mL). The mixture was stirred at room temperature for ~10 mins before sealing in a stainless-steel autoclave and placing in a solvothermal oven at 220 °C for 72 hours. The autoclave was removed and allowed to cool naturally to room temperature. The solids were collected by centrifuge and washing with water (1 x 100 mL), DMF (2 x 100 mL), then ethanol (2 x 100 mL), before drying in a desiccator under vacuum at room temperature overnight.

MIL-53(Cr)-Br: 2-Bromoterephthalic acid (2.45 g, 1 mmol) and chromium (III) chloride hexahydrate (2.66 g, 1 mmol) were added to a 200 mL Teflon autoclave liner and water (100 mL) was added along with 12 M HCl (1.5 mL). The mixture was stirred at room temperature for ~10 mins before sealing in a stainless-steel autoclave and placing in a solvothermal oven at 220 °C for 72 hours. The autoclave was removed and allowed to cool naturally to room temperature. The solids were collected by centrifuge and washing with water (1 x 100 mL), DMF (2 x 100 mL), then ethanol (2 x 100 mL), before drying in a desiccator under vacuum at room temperature overnight.

MIL-53(Cr)-NO_2: 2-Nitroterephthalic acid (1.055 g, 0.5 mmol) and chromium (III) chloride hexahydrate (1.33 g, 0.5 mmol) were added to a 200 mL Teflon autoclave liner and water (50 mL) was added along with 12 M HCl (0.5 mL). The mixture was stirred at room temperature for ~10 mins before sealing in a stainless-steel autoclave and placing in a solvothermal oven at 220 °C for 72 hours. The autoclave was removed and allowed to cool naturally to room temperature. The solid was collected by centrifuge and washing with water (1 x 50 mL), DMF (2 x 50 mL), then ethanol (2 x 50 mL), before drying in a desiccator under vacuum at room temperature overnight.

Activation protocol

To exchange the pore-bound terephthalic acid, the samples were treated in DMF (100 mL/g) at 220 °C overnight in a stainless-steel autoclave. The solid was collected by centrifuge and washed once with DMF, then dried in a desiccator under vacuum at room temperature. This yielded a product with bound DMF, MIL-53(Cr)-X-DMF.[S3] DMF was removed from the sample by refluxing in methanol (100 mL/g) overnight in an RBF and then washing with methanol (2 x 100 mL/g), before drying in a desiccator under vacuum at room temperature overnight.

S3. Powder X-Ray Diffraction Analyses

S3.1. Indexing

Where possible, powder X-ray diffractograms were indexed and/or Pawley fits were obtained. A comparison of all crystallographic data obtained with some pertinent literature examples is given in Table S1.

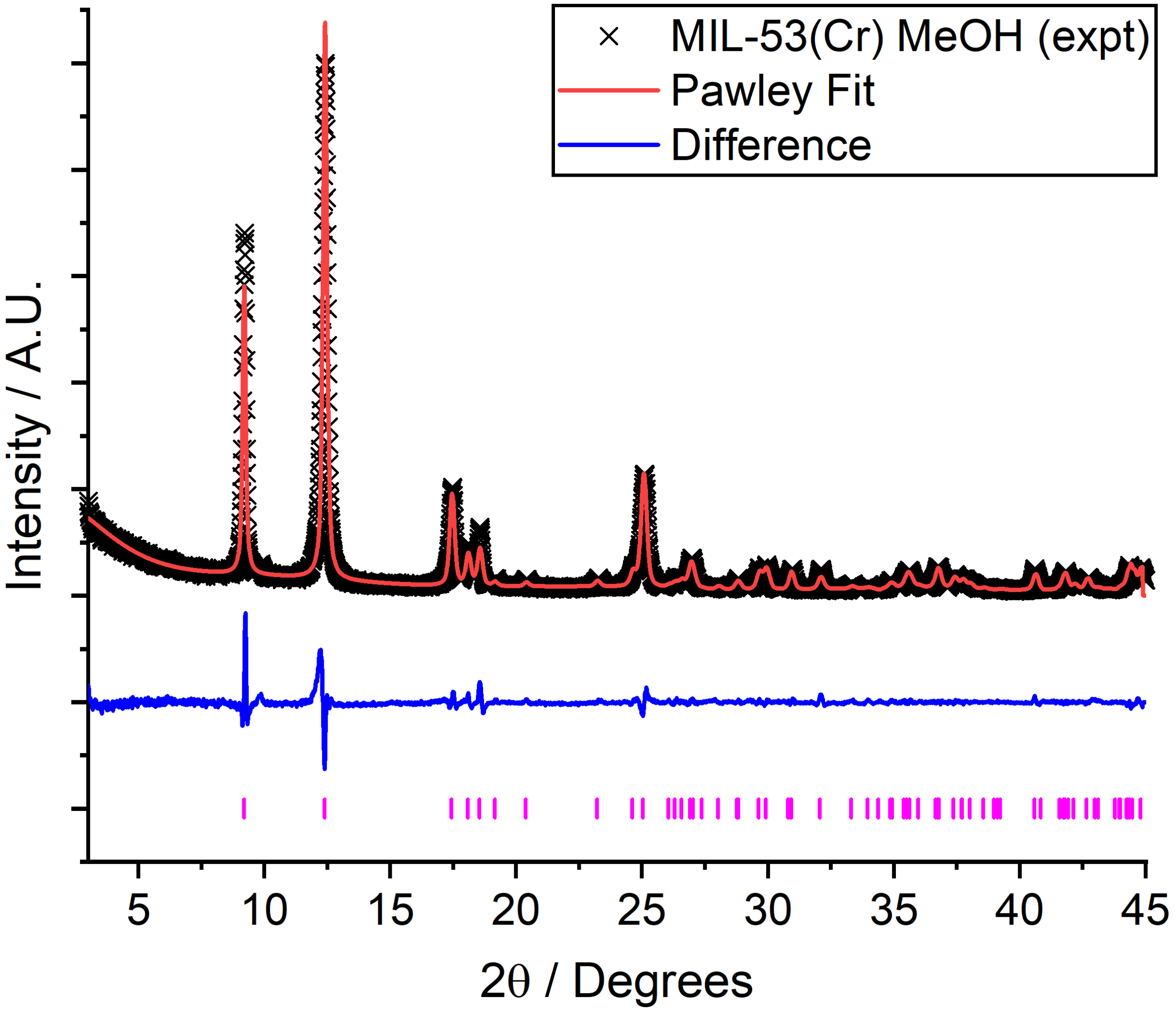

Figure S1. Pawley fitting (R_{wp} = 12.6%) of powder X-ray diffractogram of MIL-53(Cr) MeOH.

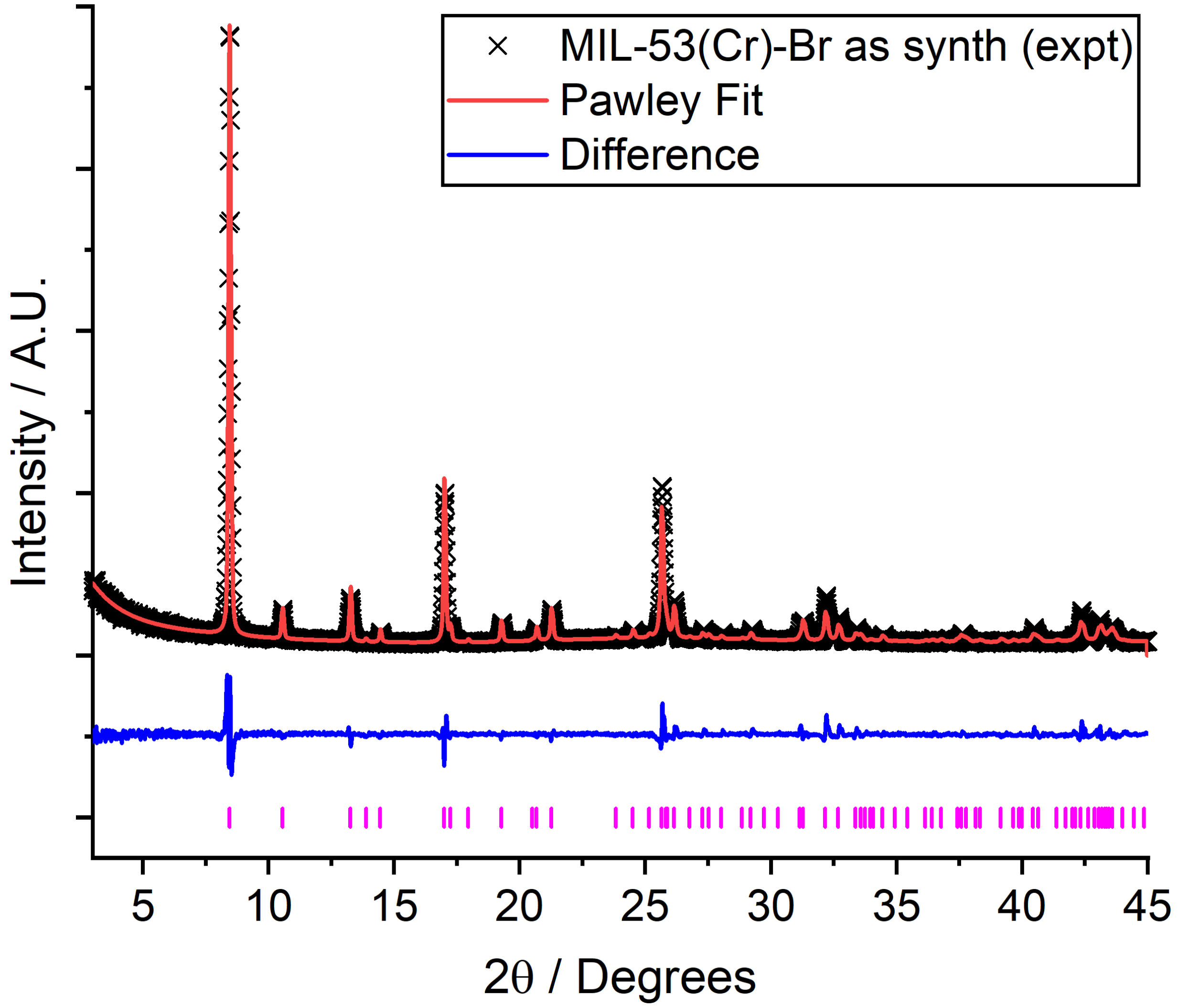

Figure S2. Pawley fitting (R_{wp} = 10.83%) of powder X-ray diffractogram of MIL-53(Cr)-Br as synthesised.

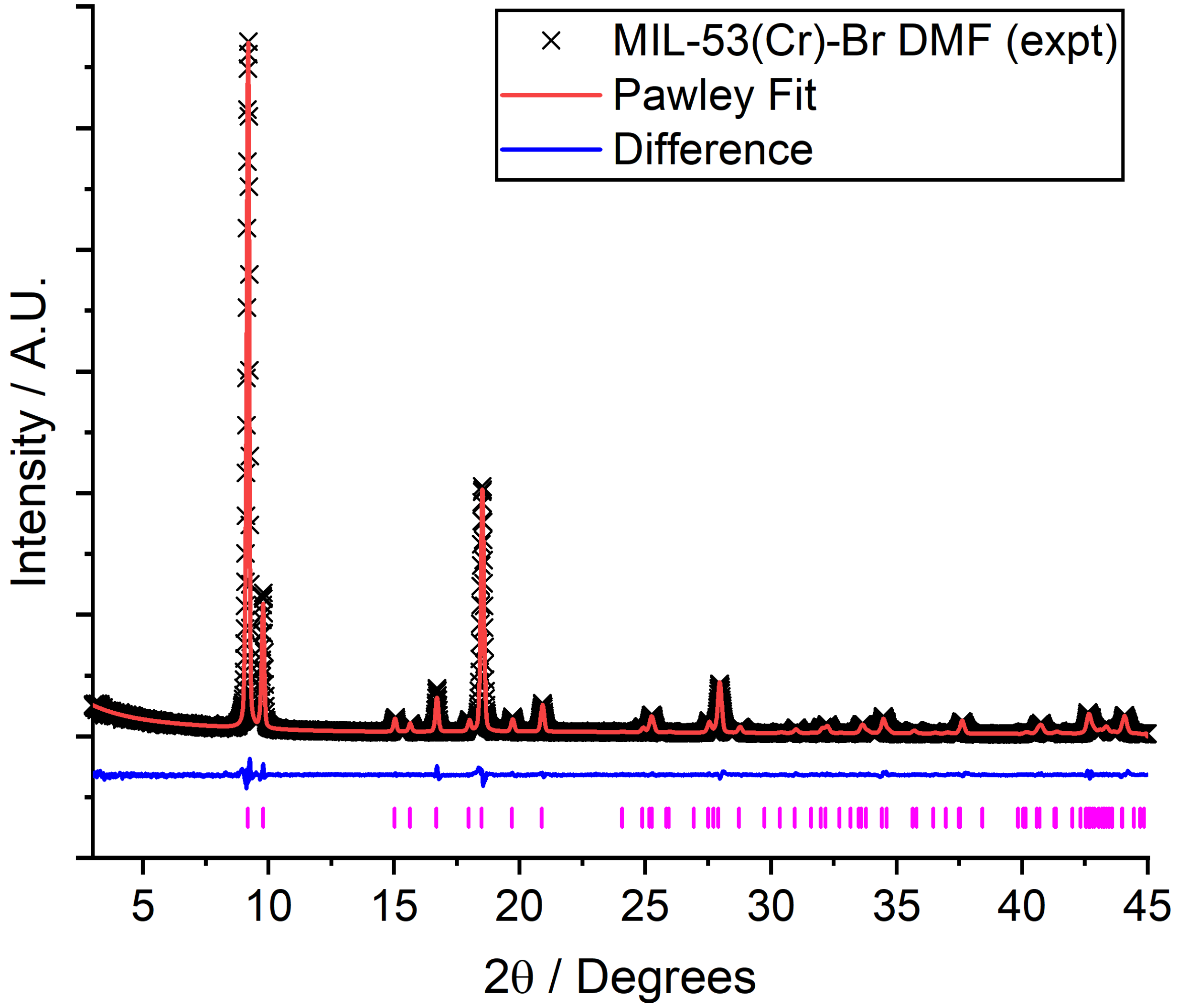

Figure S3. Pawley fitting (R_{wp} = 8.24%) of powder X-ray diffractogram of MIL-53(Cr)-Br DMF.

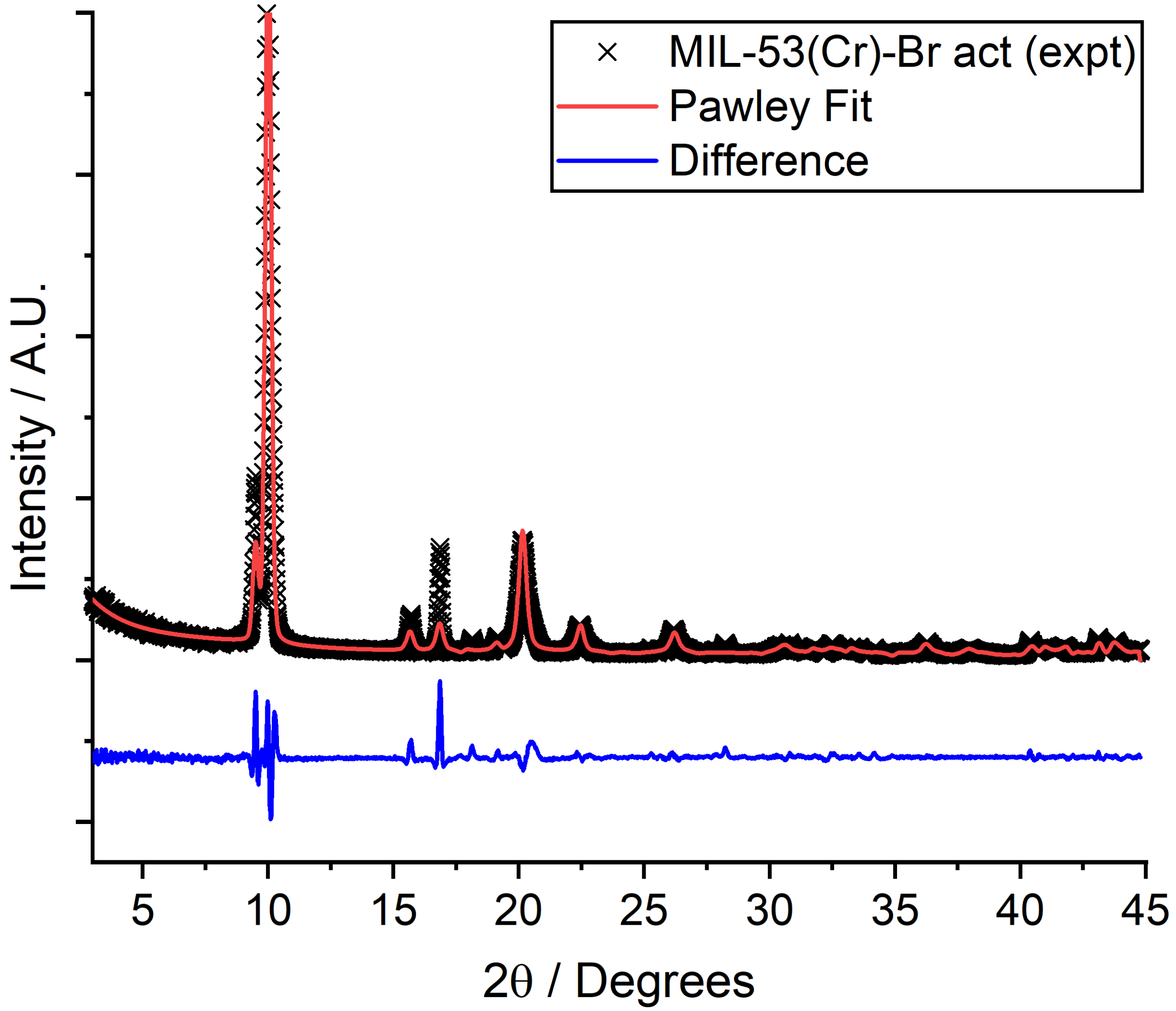

Figure S4. Pawley fitting (R_{wp} = 15.86%) of powder X-ray diffractogram of MIL-53(Cr)-Br activated.

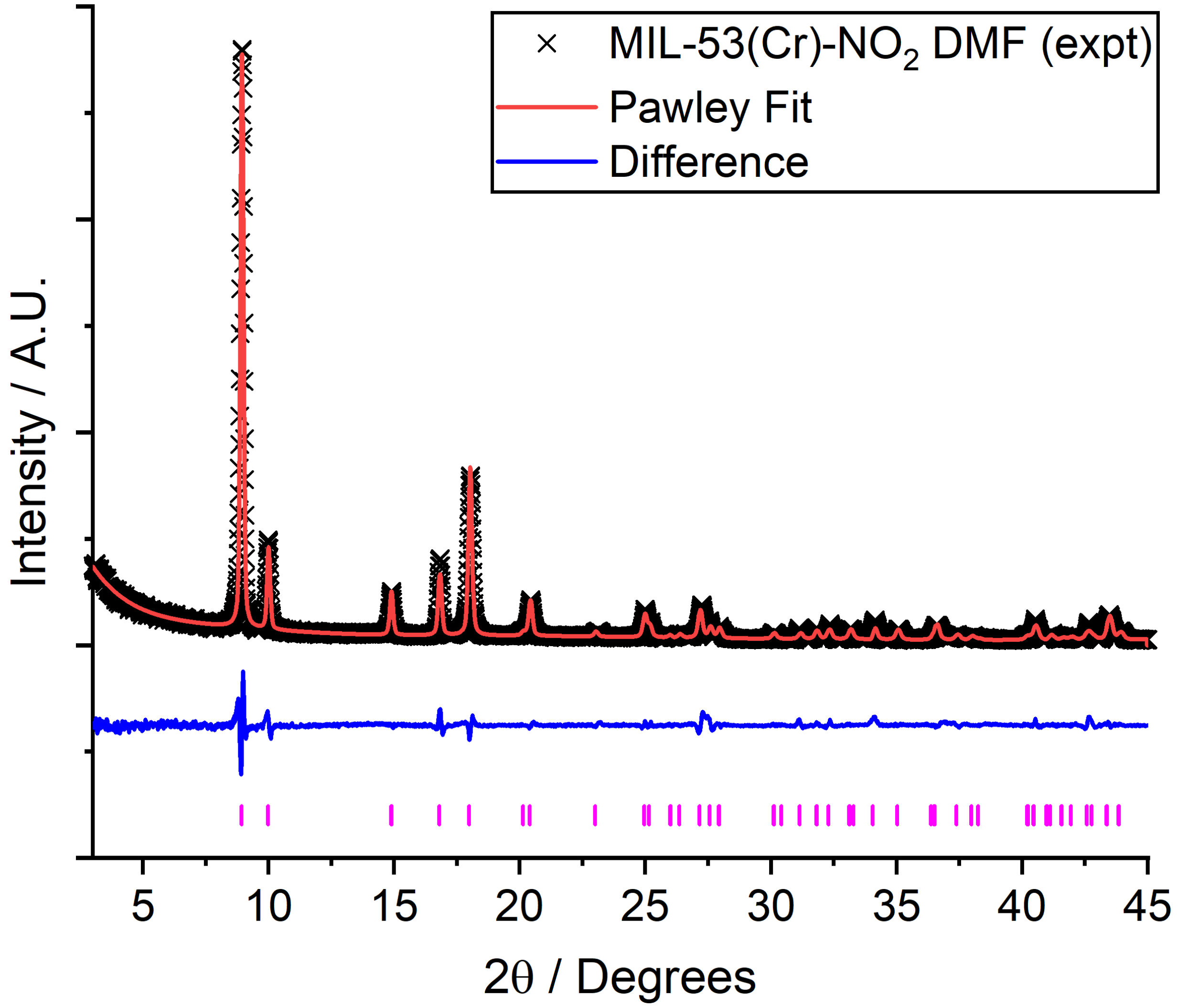

Figure S5. Pawley fitting (R_{wp} = 11.22%) of powder X-ray diffractogram of MIL-53(Cr)-NO_2 DMF.

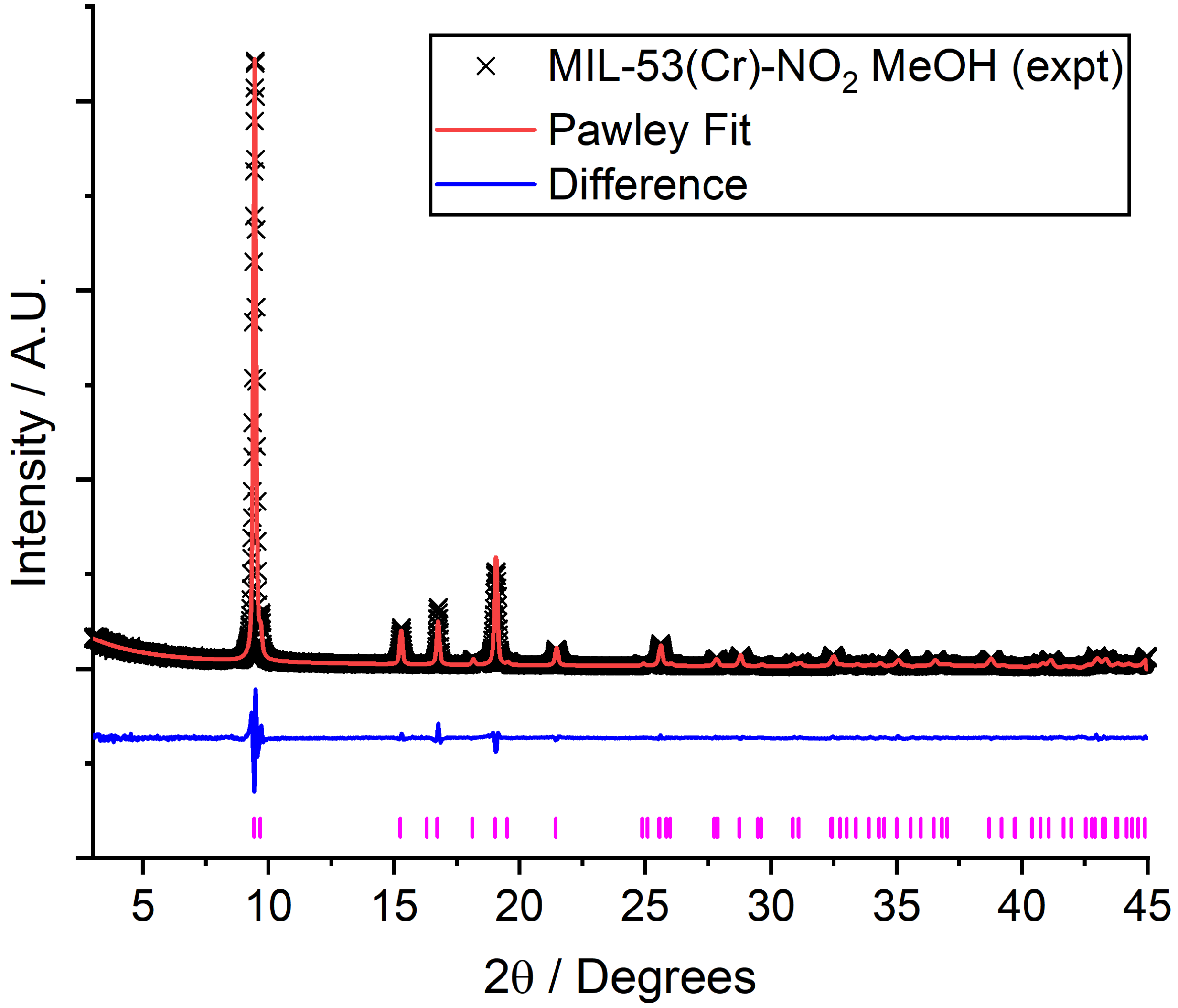

Figure S6. Pawley fitting (R_{wp} = 10.63%) of powder X-ray diffractogram of MIL-53(Cr)-NO_2 MeOH.

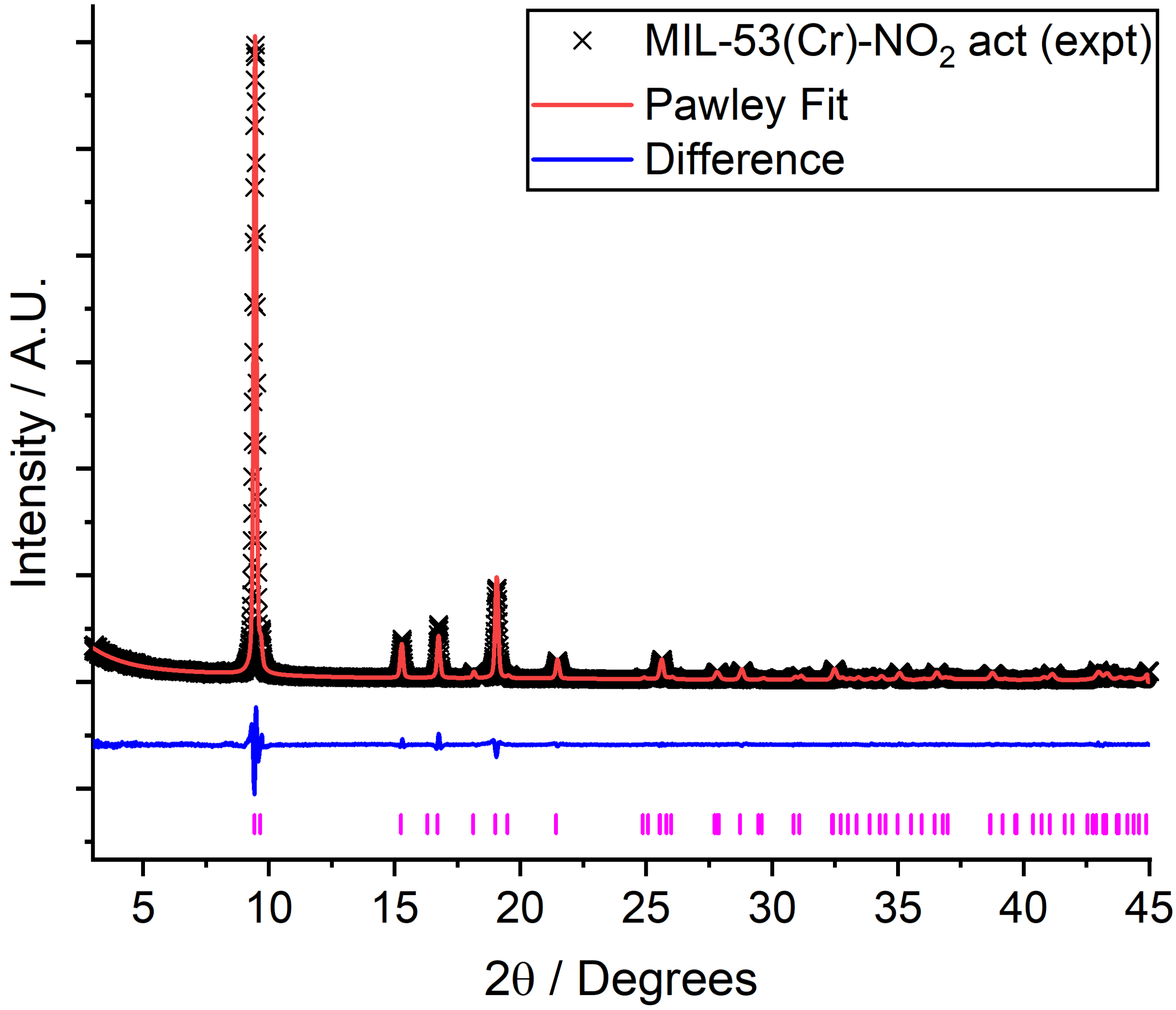

Figure S7. Pawley fitting (R_{wp} = 9.73%) of powder X-ray diffractogram of MIL-53(Cr)-NO_2 activated.

Table S1. Crystallographic parameters derived from experimental powder X-ray diffraction data in comparison with pertinent literature structures.[a]

**MOF**	**Space Group**	***a* / Å**	***b* / Å**	***c* / Å**	**α / °**	**β / °**	**γ / °**	***V* / Å³**
MIL-53(Cr) DMF	Imma	6.19471	11.46168	17.73382	90	90	90	1259.134
MIL-53(Cr) MeOH	C2/c	19.62(1)	7.624(1)	6.779(2)	90	104.3789	90	982.8(5)
MIL-53(Cr)_lt[b]	C2/c	19.685(4)	7.849(1)	6.782(1)	90	104.90(2)	90	1012.64
MIL-53(Cr)-Br as	Immm	6.8677(8)	13.291(1)	16.662(1)	90	90	90	1520.9(2)
MIL-53(Cr)-Br DMF	Imcm	6.8734(6)	11.2951(7)	17.968(1)	90	90	90	1394.9(2)
MIL-53(Cr)-Br MeOH	C2/m	11.23165	18.63846	5.92537	90	117.31	90	1102.163
MIL-53(Cr)-Br act	C2/m	10.03(2)	18.49(3)	5.662(9)	90	95.59(4)	90	1045(4)
MIL-53(In)-Br as[c]	Imma	17.4254(9)	7.2516(3)	13.3713(6)	90	90	90	1689.6(1)
MIL-53(Cr)-NO_2 as	Imcm	6.90037	13.62061	16.91884	90	90	90	1590.155
MIL-53(Cr)-NO_2 DMF	I2cm	6.824(1)	11.825(2)	17.564(3)	90	90	90	1417.2(7)
MIL-53(Cr)-NO_2 MeOH	Imcm	6.826(1)	10.811(2)	18.110(3)	90	90	90	1337(6)
MIL-53(Cr)-NO_2 act	Imcm	6.8284(8)	10.811(1)	18.126(2)	90	90	90	1338.1(4)
MIL-53(In)-NO_2 as[d]	Imma	18.6290(9)	7.3019(4)	11.4527(6)	90	90	90	1558(1)

[a]If ESDs are provided, then data comes from Pawley fits in Figures S1-S7. If no ESDs are provided, then data are from indexing only.

[b]Data taken from structure derived from room temperature powder diffraction data and computational modelling.[S4]

[C]Data taken from single crystal structure collected at 293 K.[S5]

[d]Data taken from single crystal structure collected at 293 K.[S5]

S3.2. Comparison to Literature Data

Diffractograms of each material were compared to literature data for MIL-53(Cr),[S4] MIL-53(In)-Br, and MIL-53(In)-NO_2,[S5] to confirm phase formation.

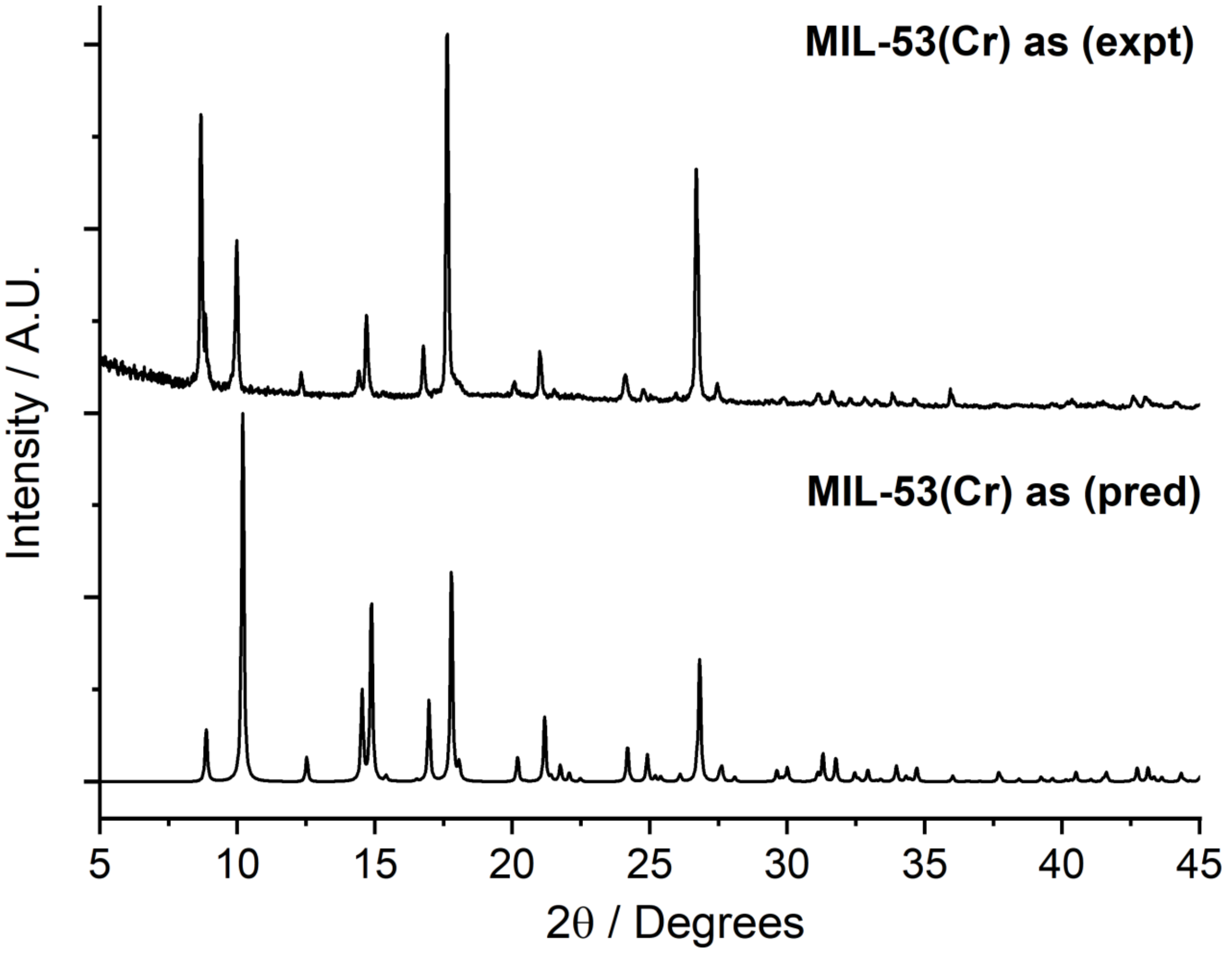

Figure S8. Comparison of powder X-ray diffractograms for as-synthesised MIL-53(Cr) and the predicted structure of MIL-53(Cr) as.[S4]

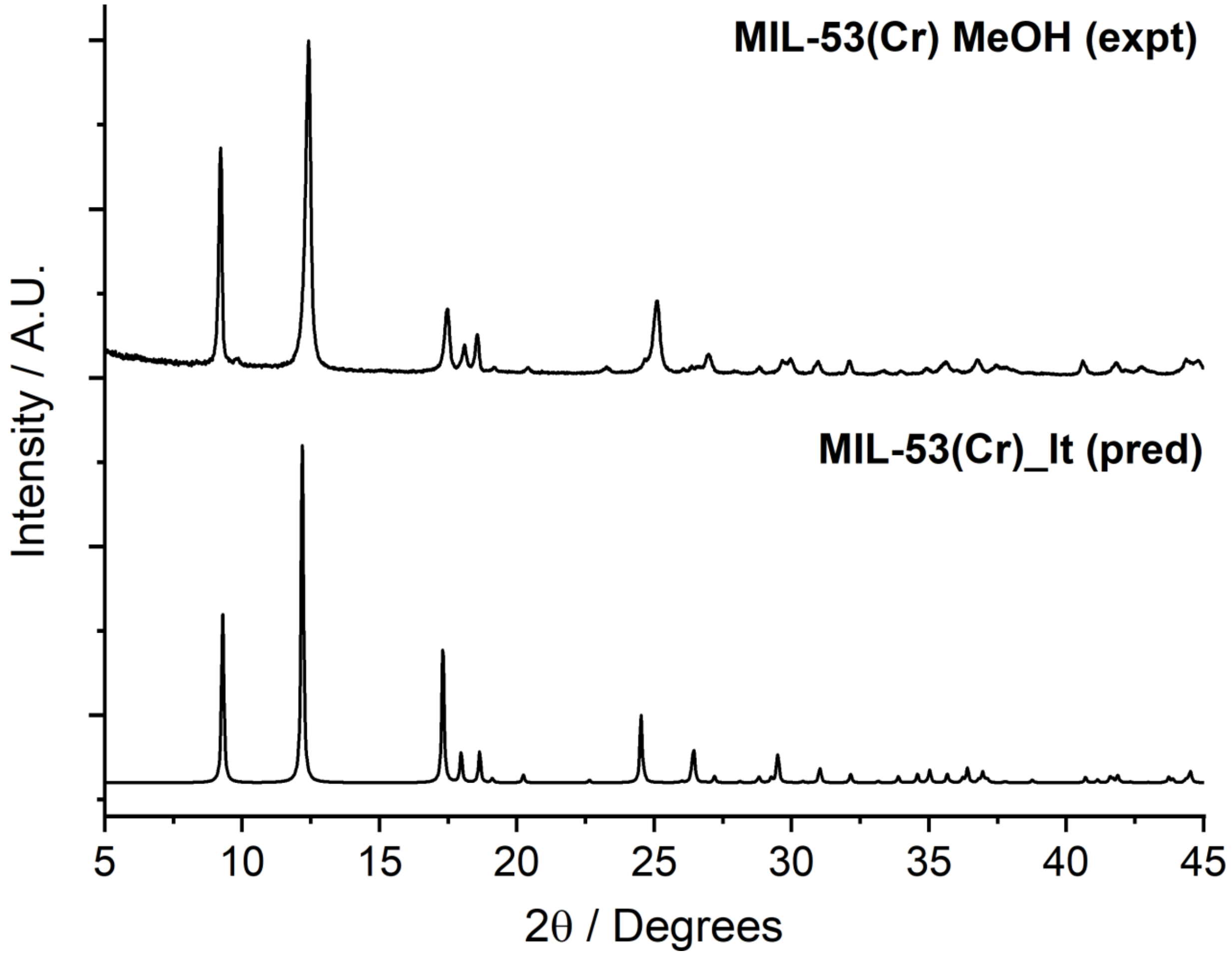

Figure S9. Comparison of powder X-ray diffractograms for MIL-53(Cr) after DMF and MeOH treatment, and the predicted structure of MIL-53(Cr)_lt.[S4]

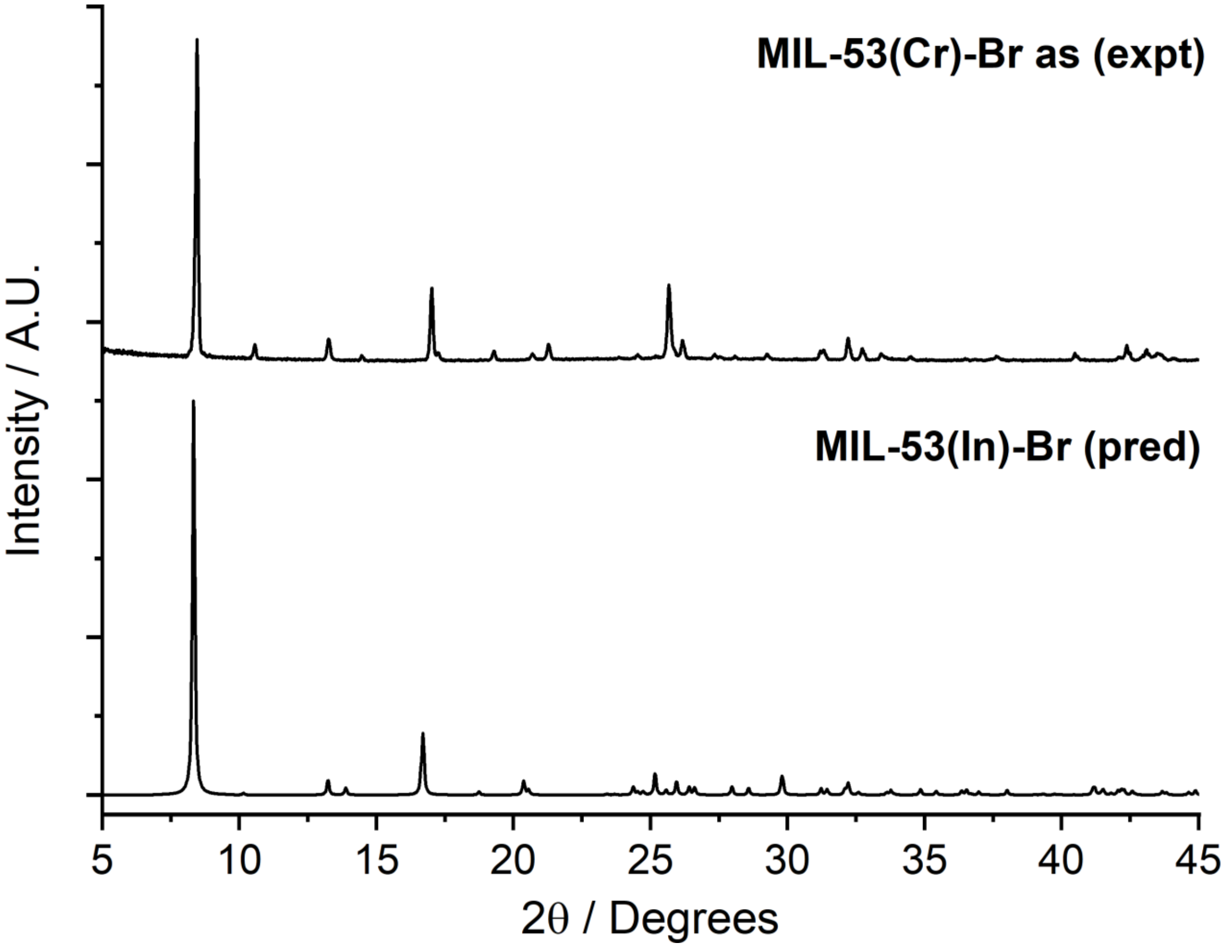

Figure S10. Comparison of powder X-ray diffractograms for as-synthesised MIL-53(Cr)-Br and that predicted from the crystal structure of MIL-53(In)-Br.[S5]

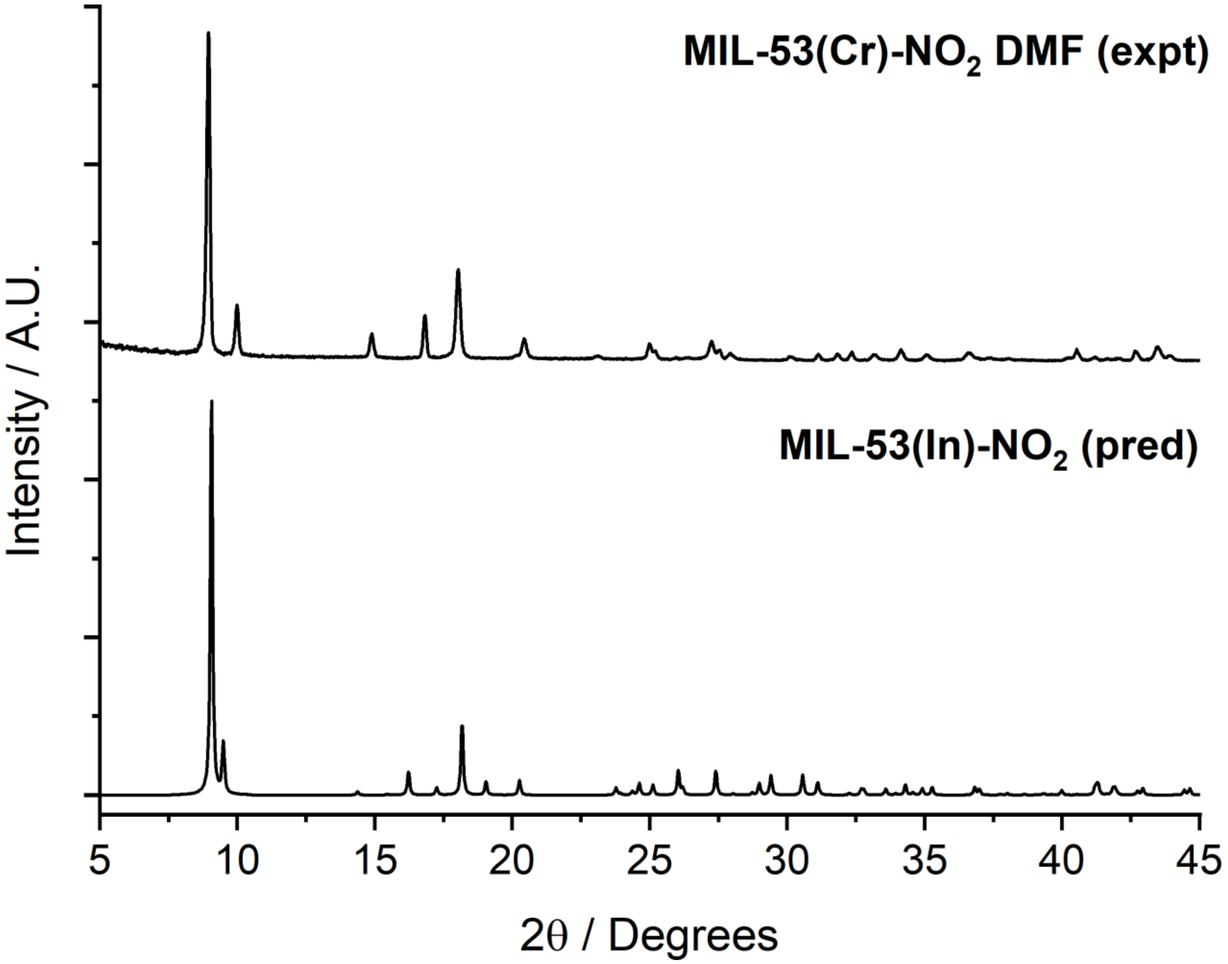

Figure S11. Comparison of powder X-ray diffractograms for DMF treated MIL-53(Cr)-NO_2 and that predicted from the crystal structure of MIL-53(In)-NO_2.[S5]

S3.3. Comparisons Across Synthesis and Work-Up

Comparisons of powder X-ray diffraction data are given for the three Cr PCPs across each stage of synthesis and work-up: as-synthesised, DMF-treated, MeOH treated, and activated. These show the structural differences induced by the functional groups.

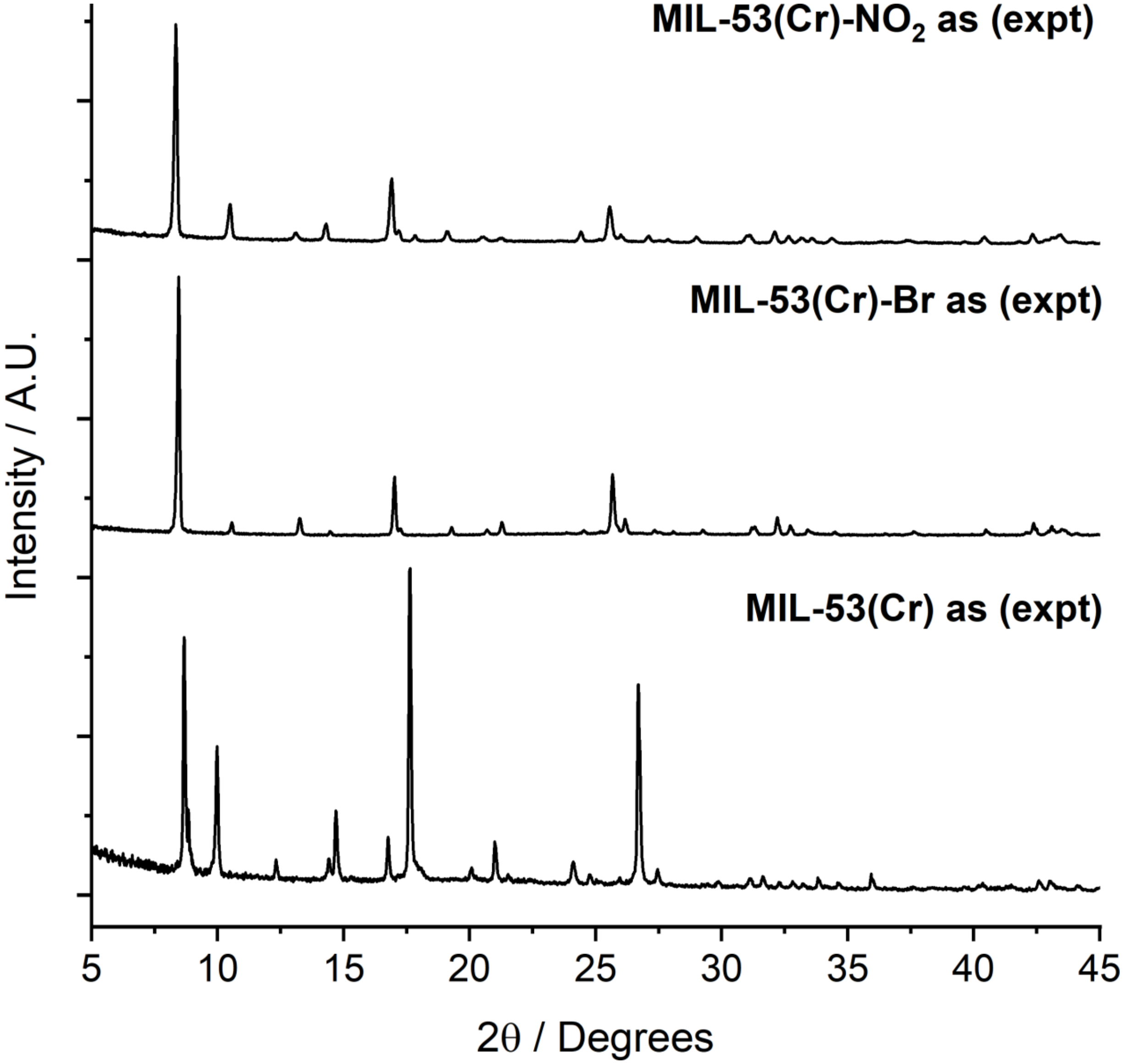

Figure S12. Comparison of powder X-ray diffractograms for as-synthesised samples of MIL-53(Cr), MIL-53(Cr)-Br, and MIL-53(Cr)-NO_2.

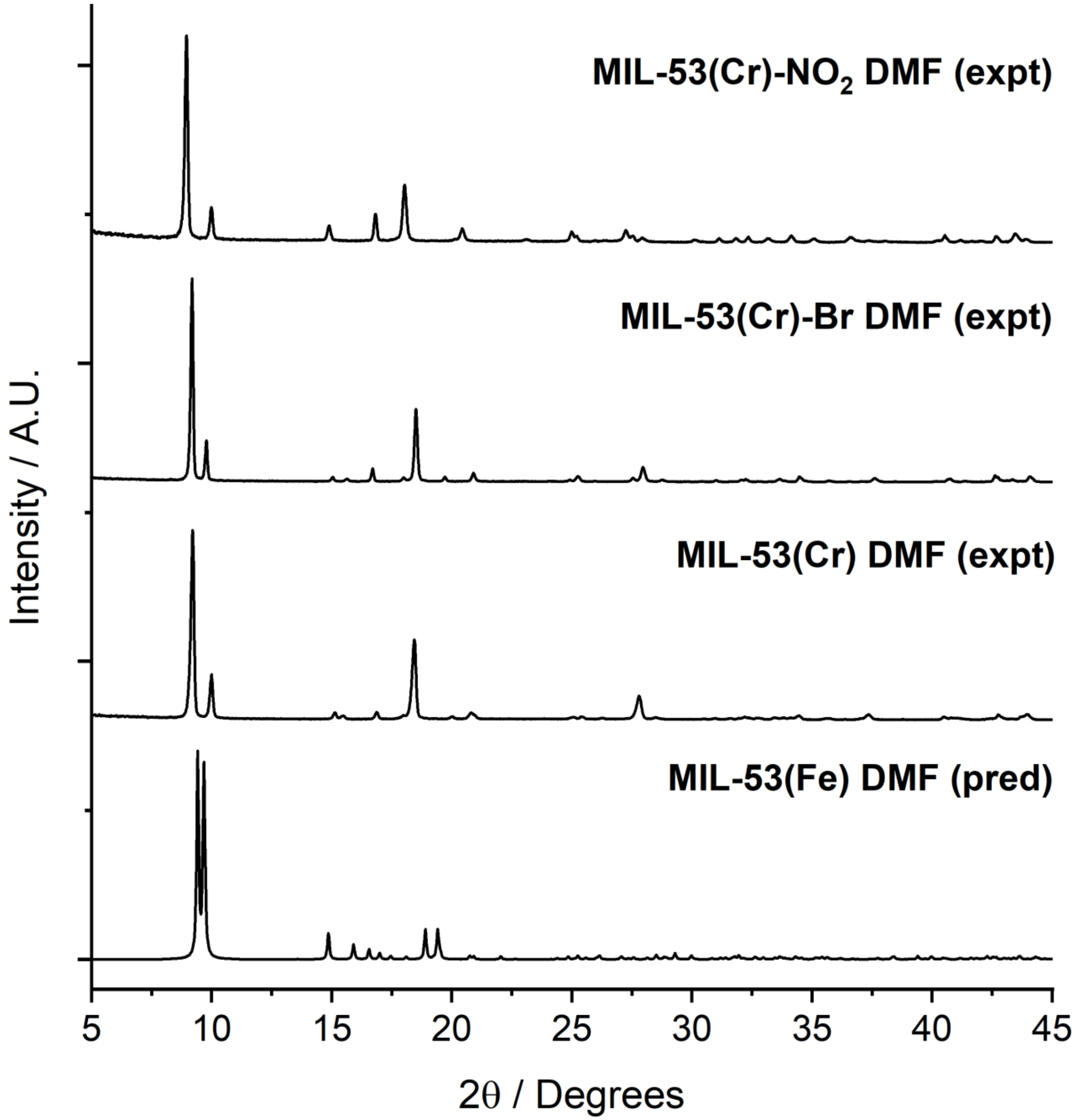

Figure S13. Comparison of powder X-ray diffractograms for DMF treated samples of MIL-53(Cr), MIL-53(Cr)-Br, and MIL-53(Cr)-NO_2, compared with that predicted from the crystal structure of the DMF solvate of MIL-53(Fe).[S6]

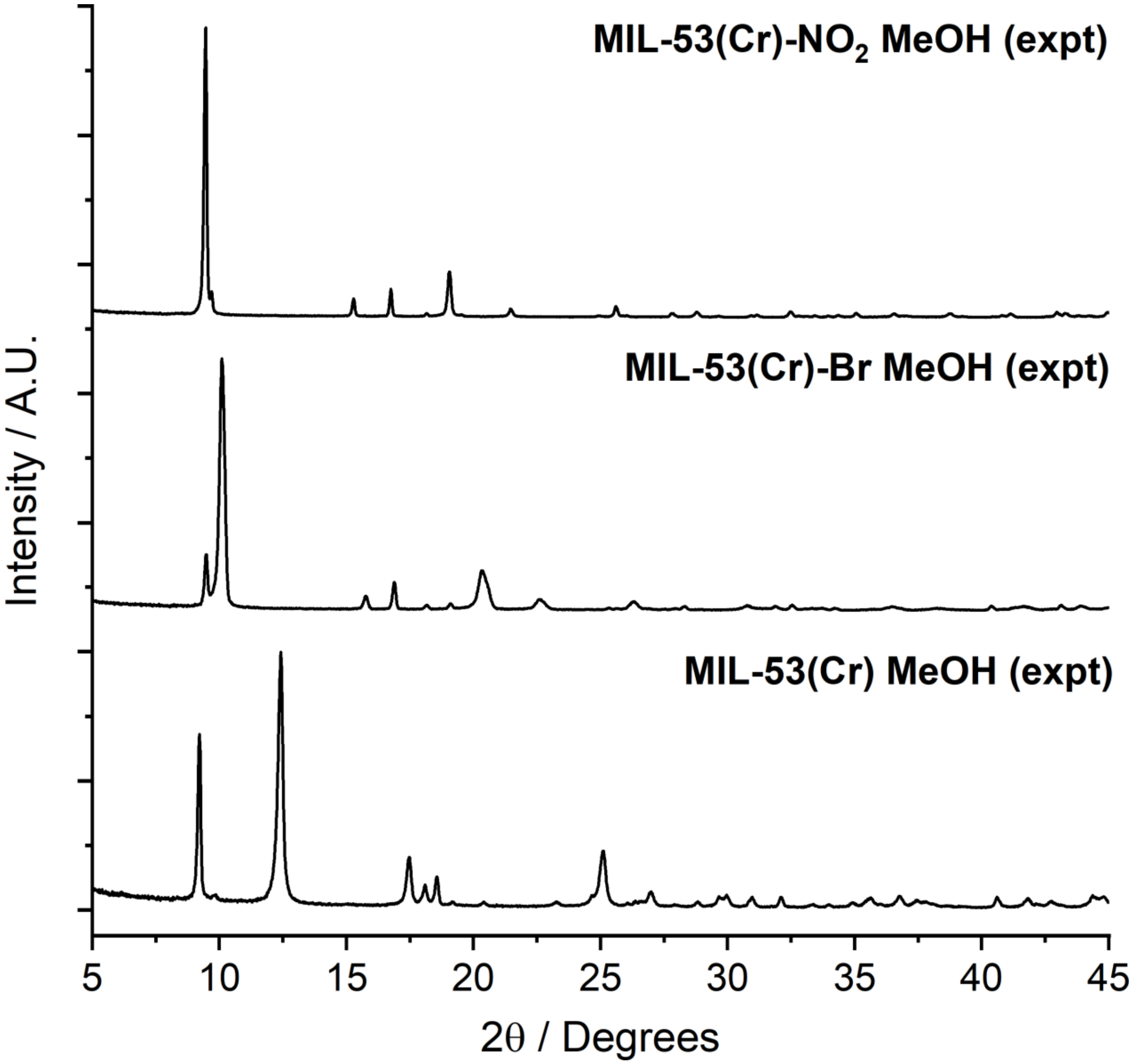

Figure S14. Comparison of powder X-ray diffractograms for DMF and MeOH treated samples of MIL-53(Cr), MIL-53(Cr)-Br, and MIL-53(Cr)-NO_2.

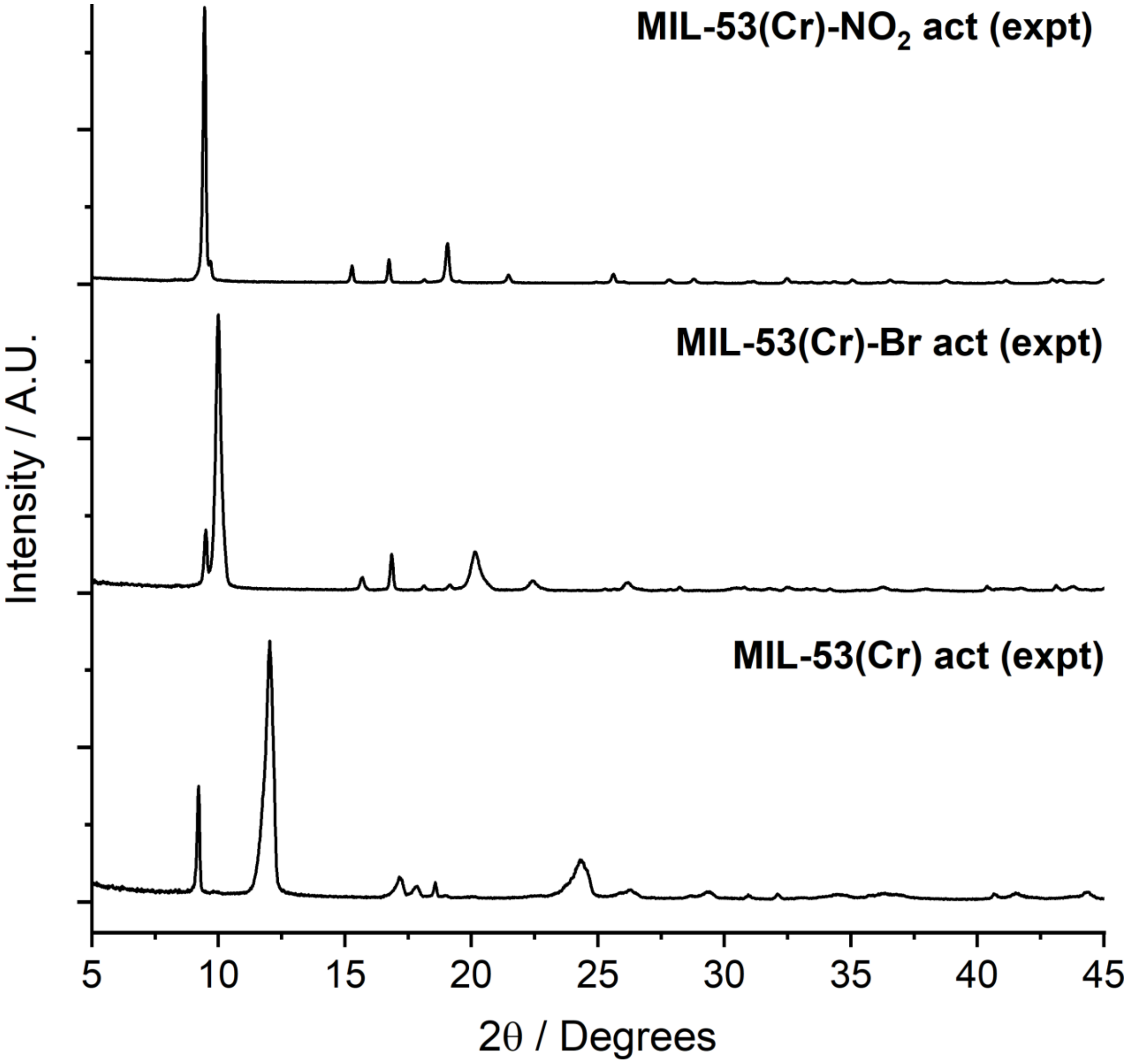

Figure S15. Comparison of powder X-ray diffractograms for activated samples of MIL-53(Cr), MIL-53(Cr)-Br, and MIL-53(Cr)-NO_2.

S4. Thermogravimetric Analysis

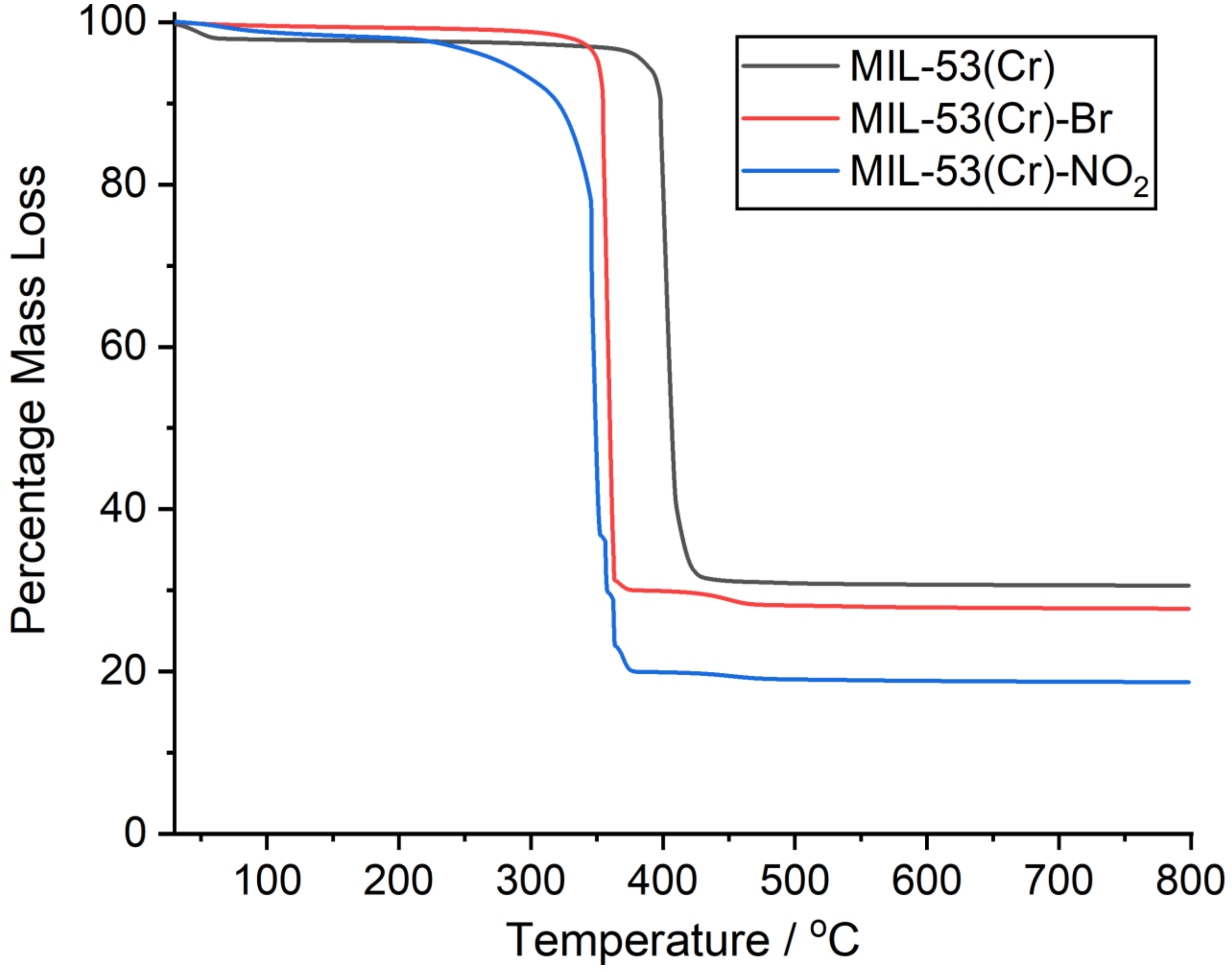

Figure S16. Comparison of thermogravimetric analysis traces for activated samples of MIL-53(Cr), MIL-53(Cr)-Br, and MIL-53(Cr)-NO_2.

S5. Adsorption Isotherms

S5.1. N_2 Adsorption Isotherms (77 K)

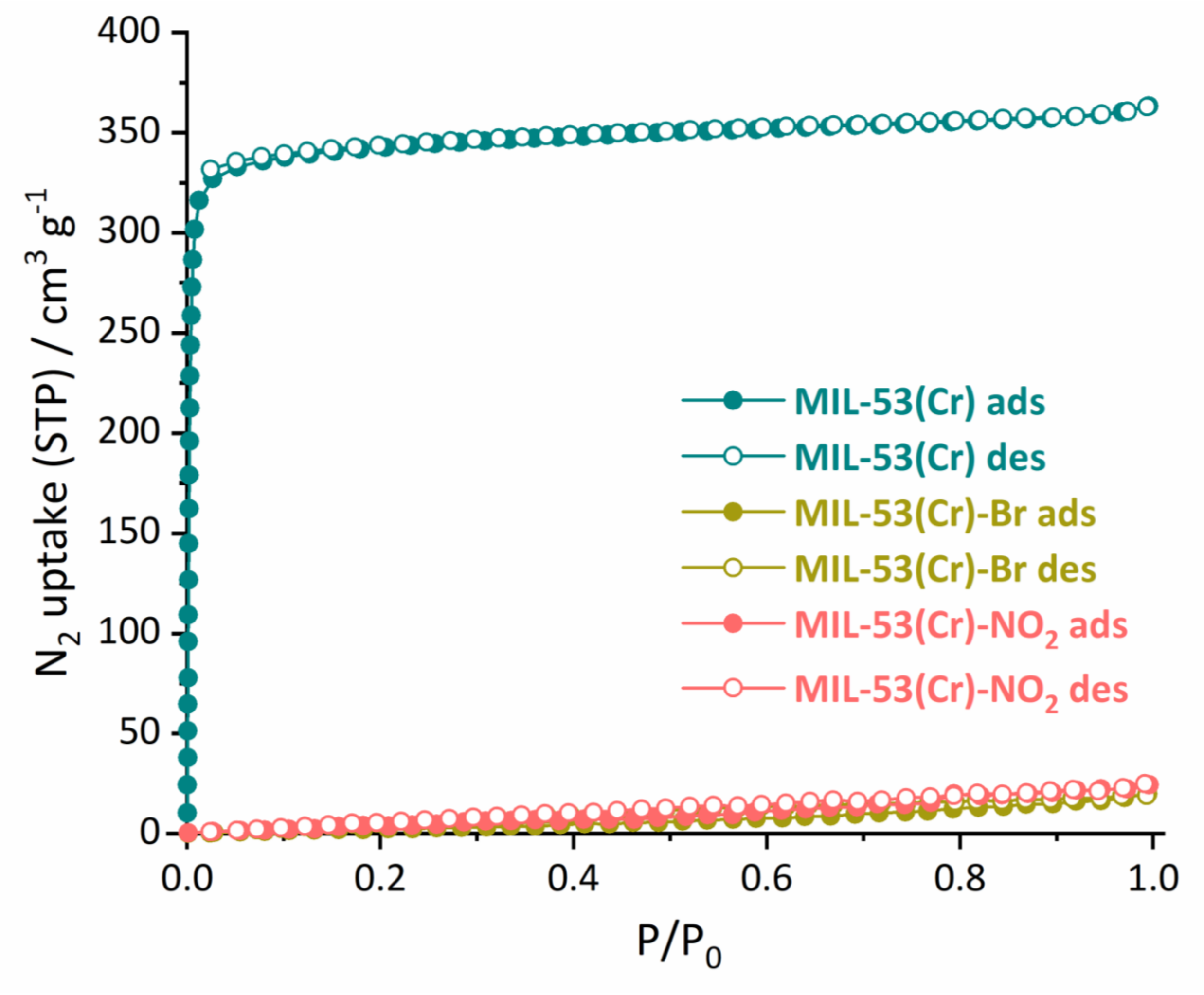

Figure S17. N_2 adsorption/desorption isotherms (77 K) of the three MIL-53(Cr) MOFs.

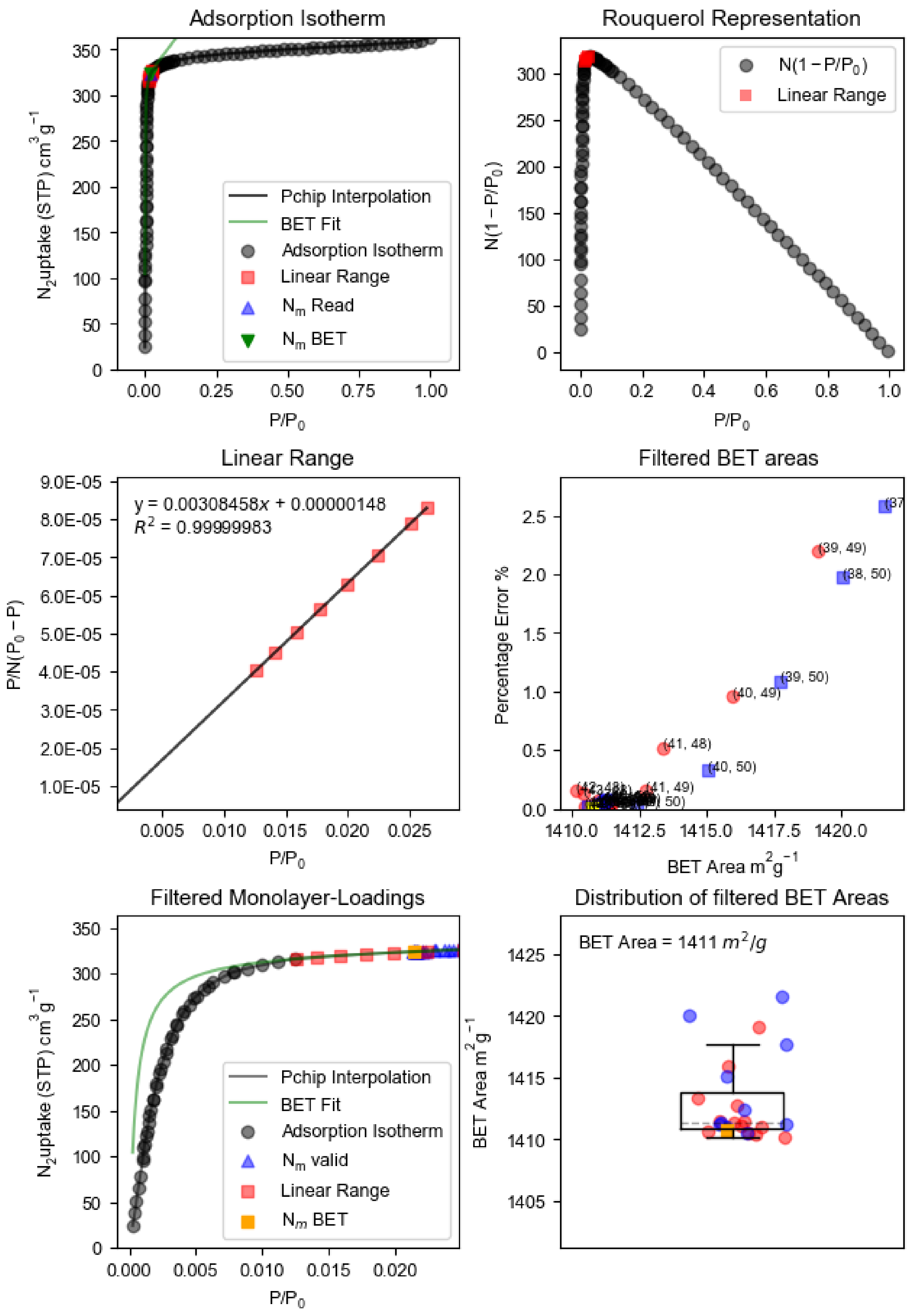

Figure S18. BETSI analysis[S2] of MIL-53(Cr) from the N_2 adsorption isotherm.

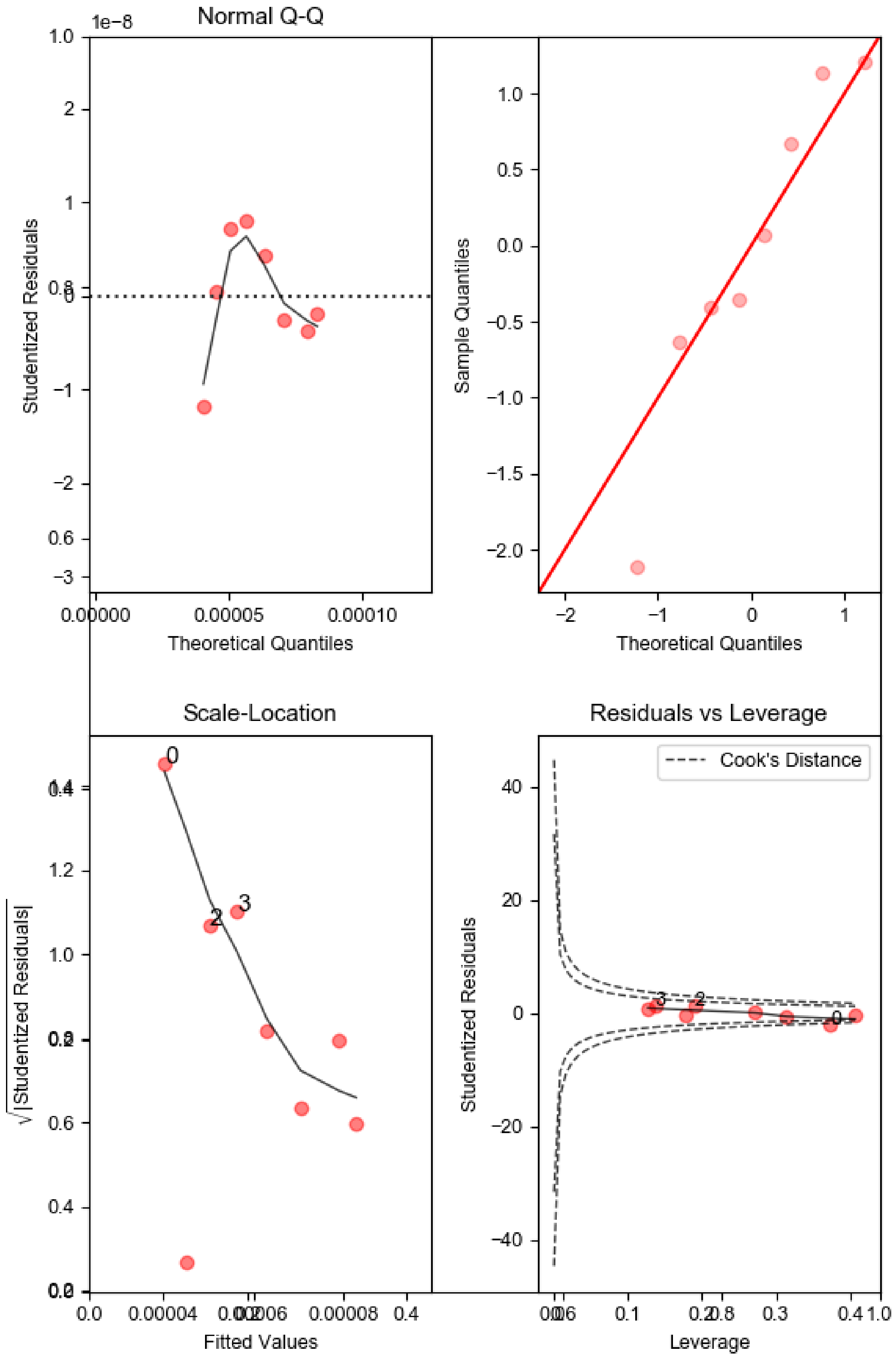

Figure S19. BETSI regression diagnostics[S2] of MIL-53(Cr) from the N_2 adsorption isotherm.

S5.2. CO_2 Adsorption Isotherms (273 K and 298 K)

CO_2 adsorption isotherms were collected at 298 K at the University of Glasgow to allow calculation of enthalpies of adsorption and direct comparison with the SO_2 adsorption data. Data collected at 273 K are presented in Figure 1d of the manuscript.

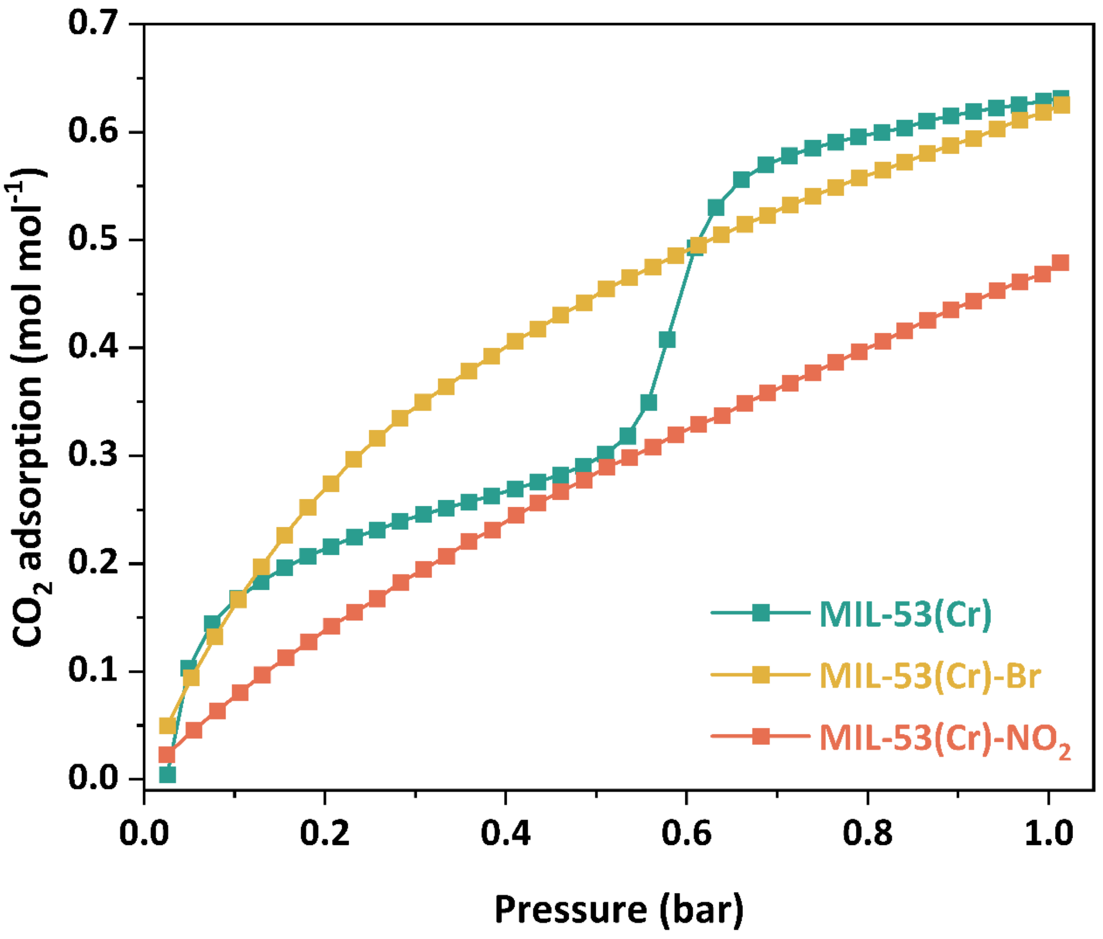

Figure S20. Experimental CO_2 adsorption–desorption isotherms for fully activated MIL-53(Cr) (blue), MIL-53(Cr)-Br (yellow), and MIL-53(Cr)-NO_2 (red) samples at 298 K and up to 1 bar.

The isosteric enthalpies of CO_2 adsorption were calculated for the MIL-53(Cr), MIL-53(Cr)-Br and MIL-53(Cr)-NO_2 materials, with the obtained values being 43.78, 27.78 and 21.89 kJ mol^{-1}, in turn.

Calculations were carried out accordingly to reported literature,[S2, S7] using a virial-type equation (Eq. S1) to fit the low coverage region of two adsorption isotherms at 273 and 298 K for the three materials (Figure S21).

$$\mathrm{Ln}(n/p) = A_0 + A_1\eta + A_2\eta^2 + \ldots \quad \text{(Eq. S1)}$$

Where p is the pressure, n is the amount adsorbed and A_0, A_1, … are the virial coefficients. The plot of Ln (n/p) give a straight line at low surface coverage (Figure S22). From the linear fittings (using the Clausius-Clapeyron equation) the virial coefficients are used to estimate the enthalpy of adsorption.

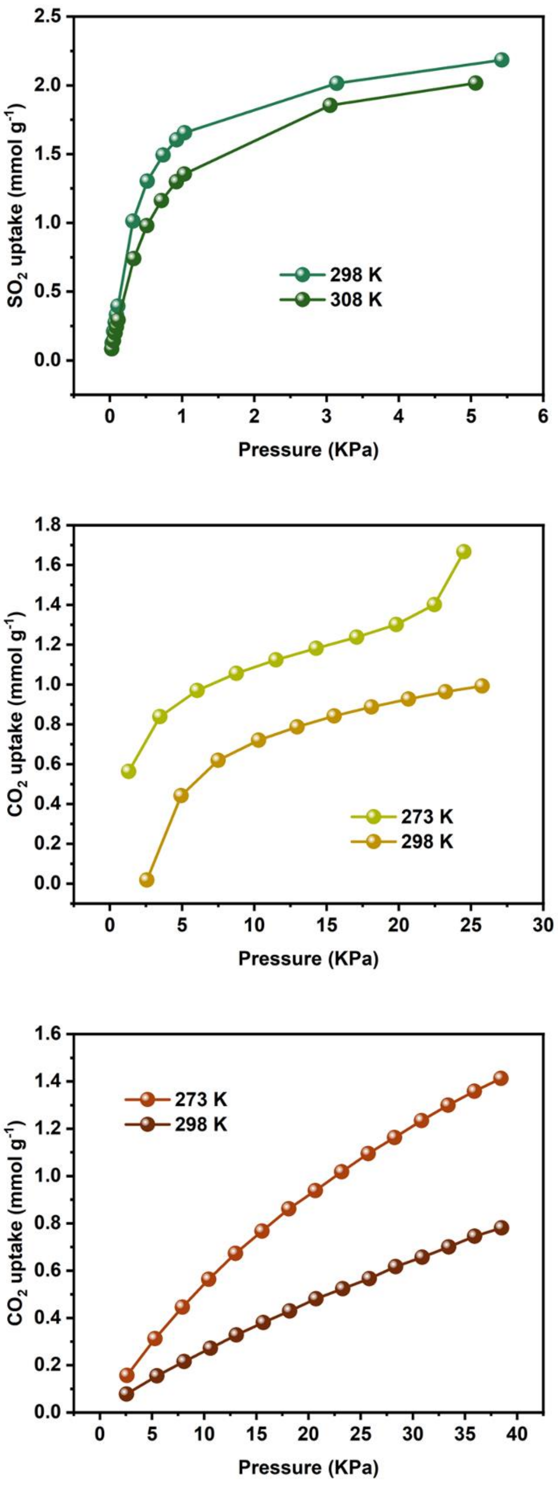

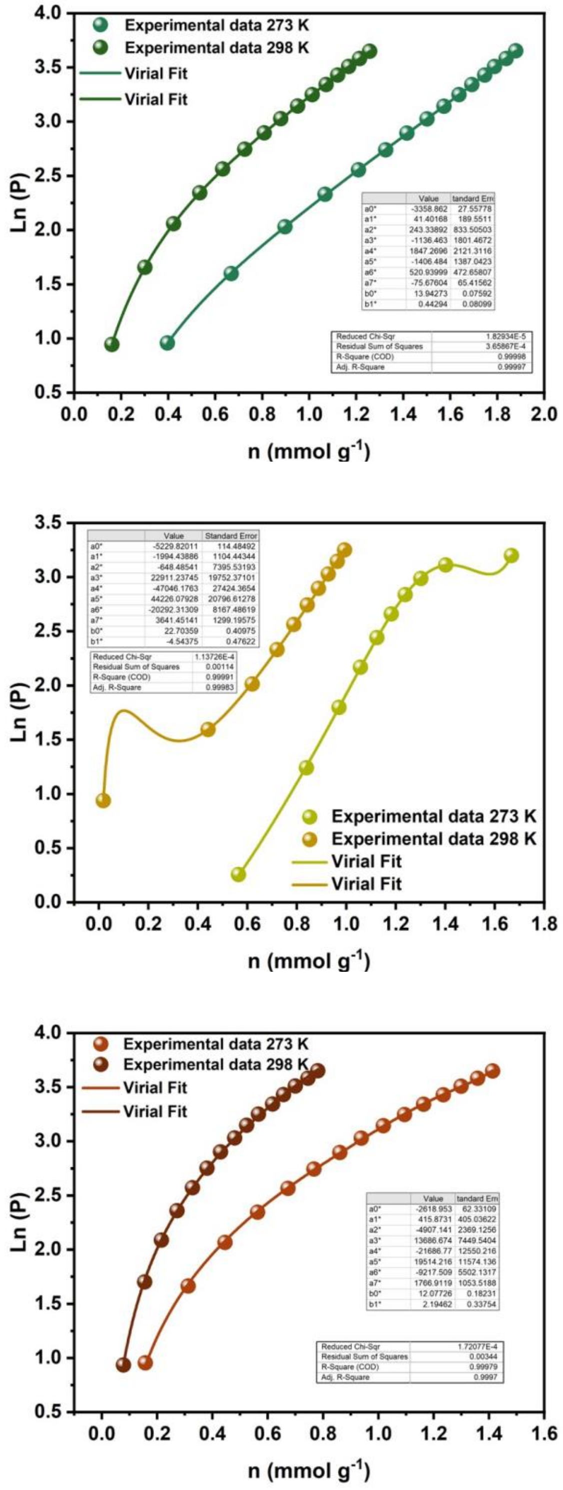

Figure S21. Experimental CO_2 adsorption isotherms for MIL-53(Cr) (green), MIL-53(Cr)-Br (yellow), and MIL-53(Cr)-NO_2 (red) samples at 273 and 298 K.

Figure S22. Virial fitting plots for the adsorption isotherms of CO_2 for MIL-53(Cr) (green), MIL-53(Cr)-Br (yellow), and MIL-53(Cr)-NO_2 (red).

S5.3. SO_2 Adsorption Isotherms (298 K)

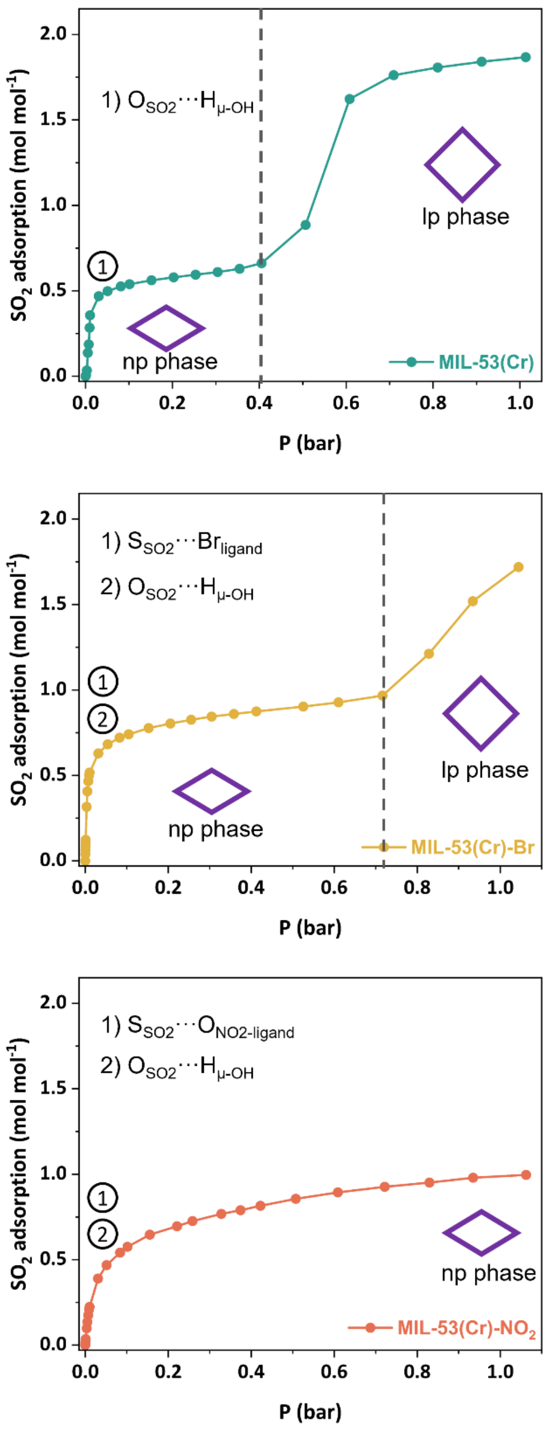

Figure S23. Schematic of the gate opening during SO_2 adsorption on MIL-53(Cr) (blue), MIL-53(Cr)-Br (yellow) and MIL-53(Cr)-NO_2 (red).

The present scheme seeks to outline the process of pore opening in our materials. It is noteworthy that it has been widely reported that upon heating MIL-53 materials, as in our activation process, they adopt the narrow pore (np) phase.[S8] In the case of MIL-53(Cr), the gate opening occurs at a gas pressure of 0.4 bar, to give way to the large pore (lp) phase. On the other hand, for MIL-53(Cr)-Br the pore opening occurs at 0.72 bar, while for MIL-53(Cr)-NO_2 the gate opening does not occur during the pressure range of our measurements (0 to 1 bar).

It should be noted that the gate opening process has been reported to depend largely on the chemical nature of the functional groups of the ligands,[S9] because these have an impact on intra-framework interactions, such as hydrogen bonds that may occur between the functional groups and the H-atom of the μ_2-OH bridging ligands from the secondary building unit of the materials. In addition, steric hindrance of the ligand functionalization modifies the inclination of the phenyl ring, which prevents rotation of the ligand and hardens the pore.[S9] That is why for MIL-53(Cr) which has no functionalization, gate opening occurs at lower pressures, while for MIL-53(Cr)-Br gate opening occurs at higher pressures, due to $Br_{ligand}\cdots H_{\mu OH}$ intra-framework interactions and Br steric hindrance hindering the opening of the pore. Meanwhile, MIL-53(Cr)-NO_2 shows no gate opening from 0 to 1 bar SO_2, suggesting that the intra-framework $O_{NO2}\cdots H_{\mu OH}$ hydrogen bonds are stronger than those occurring in the brominated material, in addition to the fact that the NO_2 group is larger than the Br group, which further impedes the rotation of the ligand and consequently prevents the pore opening.

The organic functionalization of the ligands is a key player in the SO_2 adsorption process. Based on our previous work,[S10] we propose that in the non-functionalized MIL-53(Cr) the initial jump in adsorption occurs because hydrogen bonds are forming between SO_2 molecules via their O-atoms and the H-atom of the μ-OH bridging ligand ($O_{SO2}\cdots H_{\mu\text{-}OH}$) in the np phase, these SO_2/μ-OH interactions remain predominant throughout the

adsorption, even in the lp phase, though they form with slightly longer distances. On the other hand, for the MIL-53(Cr)-Br material we propose that, in addition to hydrogen bonds, the initial adsorption is due to strong electrostatic interactions between the halogen and the S-atom of SO_2 ($S_{SO2}\cdots Br_{ligand}$), which causes the first step in the adsorption isotherm to be larger than that of the non-functionalized material.[S11] For MIL-53(Cr)-NO_2, we propose that polar interactions occur between the O-atom of the NO_2 group and the S-atom of SO_2 ($S_{SO2}\cdots O_{NO2\text{-}ligand}$), as described in other works,[S12] in addition to SO_2/μ-OH hydrogen bonds. For all three materials, in addition to the interactions already described, additional interactions between SO_2 and the organic ligands ($O_{SO2}\cdots C_{ligand}$) and interactions between the SO_2 molecules adsorbed inside the pores ($S_{SO2}\cdots O_{SO2}$) are also expected to occur.[S10]

It is worth mentioning that for MIL-53(Cr) and MIL-53(Cr)-Br a hysteresis is observed after desorption, this is in good correlation with Schneeman et al.,[S13] who postulates that upon gas adsorption there is an energy penalty associated with an increase in interfacial area as the phase change from np to lp occurs, however, for the desorption, which is the reverse structural change from lp to np it is not necessary to overcome such an energy barrier. Finally, in the case of MIL-53-NO_2 there is no hysteresis observed because the pressure necessary to achieve gate opening is not reached.

S6. Custom *ex situ* SO_2 Adsorption System

The system (Figure S4) contains two principal parts: SO_2 gas generator (A) dropping funnel with H_2SO_4 conc. [1] connected to a Schlenk flask with Na_2SO_3 (s) under stirring [2]; and the saturation chamber (B), constructed from a round flask with distilled water [3], connected to a sintered glass filter adapter [4] and to a vacuum line [5]. To begin with, a sample of about 60 mg in a 1.5 mL glass vial was activated in a sand bath at 220°C under vacuum for 24 h. Then, the vial was quickly placed on the glass adapter, and the system was evacuated with a vacuum line. Next, SO_2 gas was generated by dripping concentrated sulfuric acid over Na_2SO_3, which passed through the sample continuously for 3 hours.

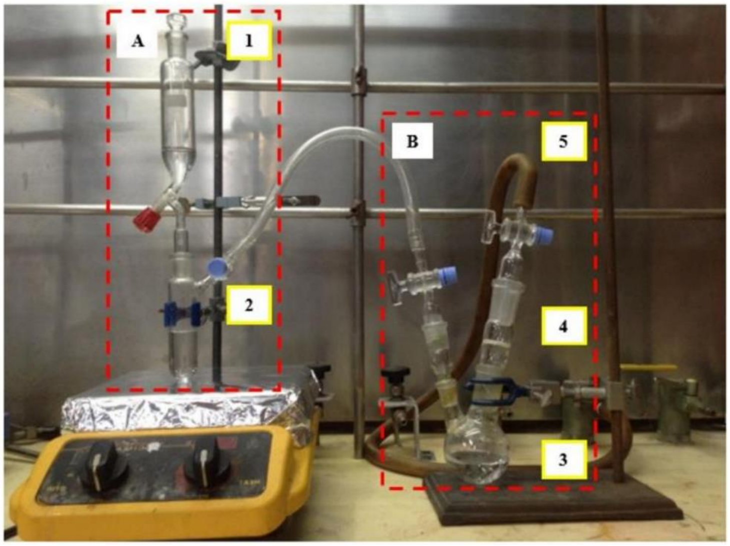

Figure S24. Ex situ SO_2 homemade system.

S7. Characterisation after SO_2 Adsorption

Samples were analysed by powder X-ray diffraction and infrared spectroscopy after exposure to SO_2.

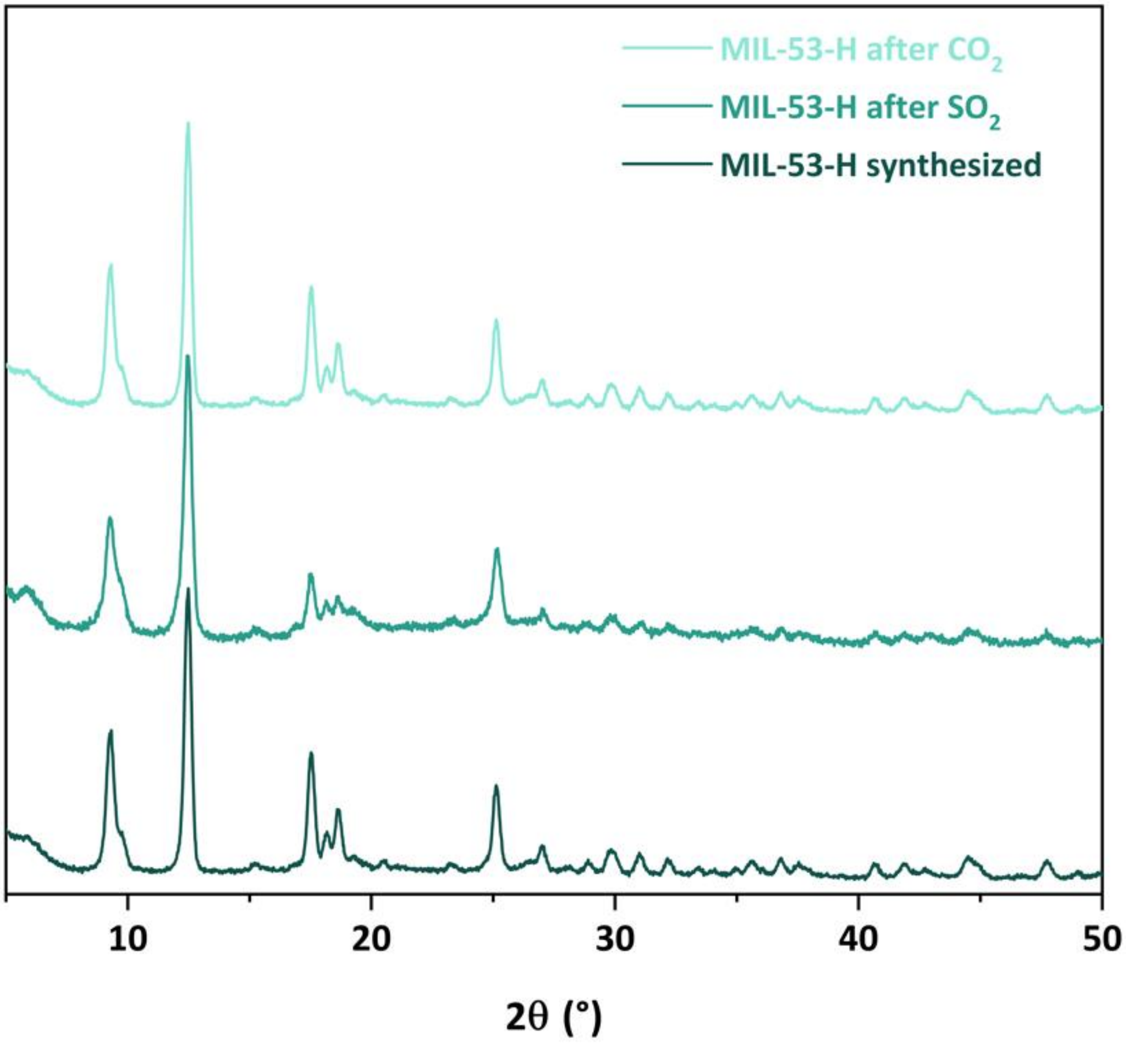

Figure S25. Comparison of the PXRD patterns of synthesized and washed MIL-53(Cr) material (bright blue), the one exposed to SO_2 (blue), and the one exposed to CO_2 (blue).

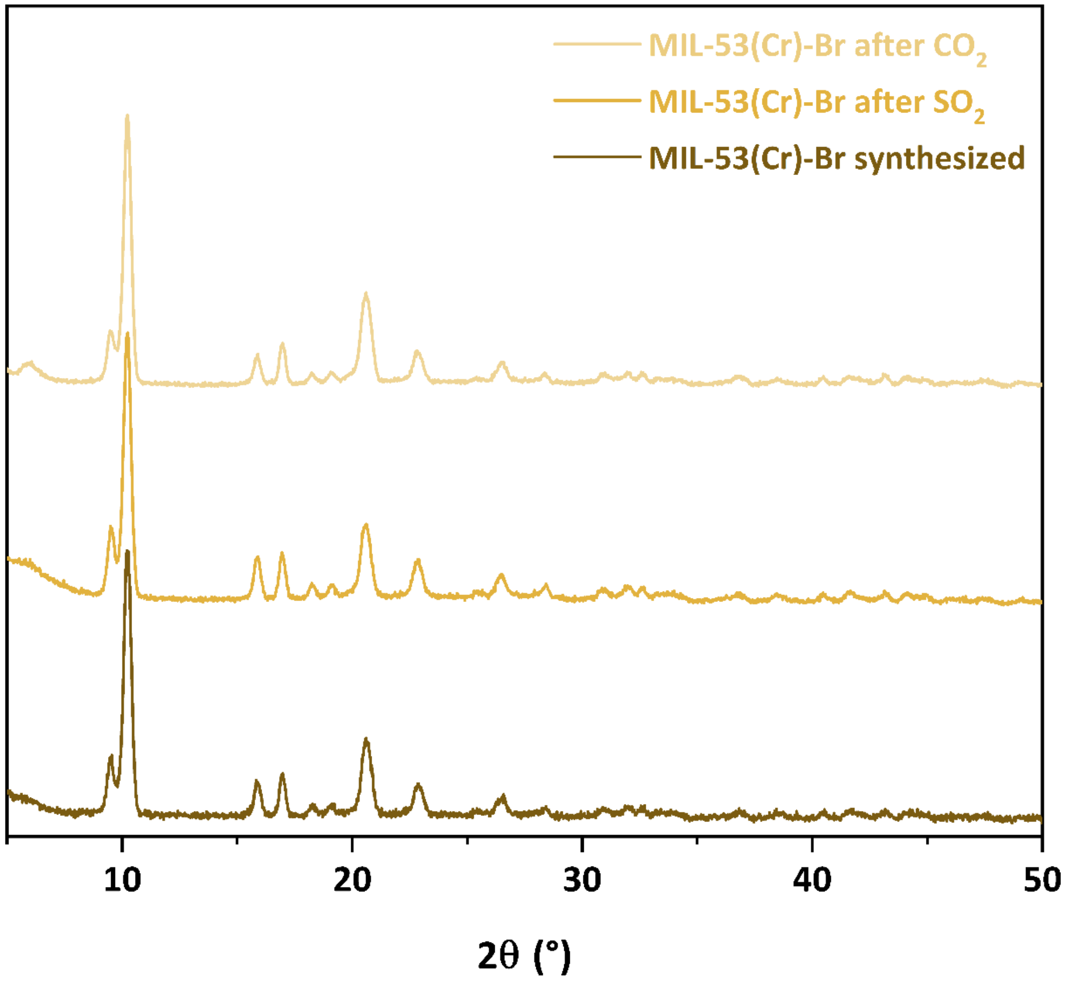

Figure S26. Comparison of the PXRD patterns of synthesized and washed MIL-53(Cr)-Br material (brown), exposed to SO_2 (mustard), and exposed to CO_2 (yellow).

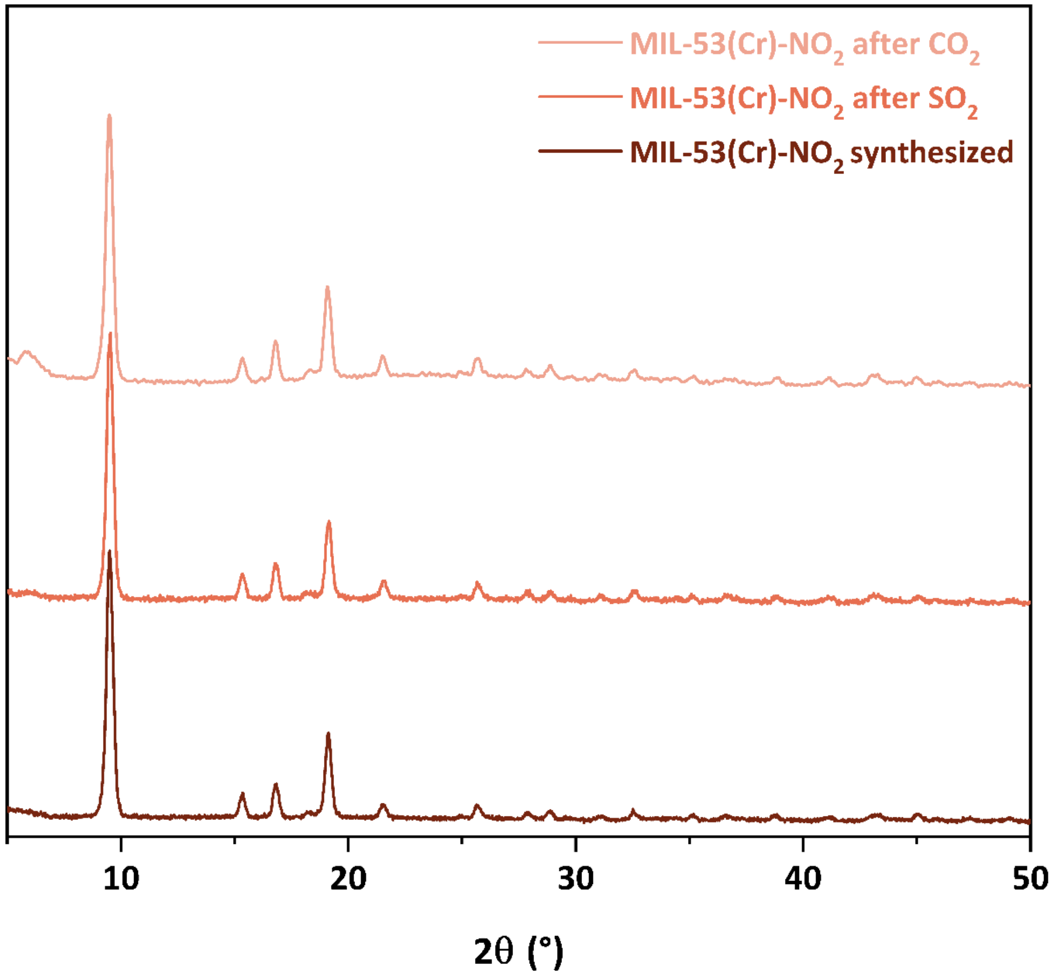

Figure S27. Comparison of the PXRD patterns of synthesized and washed MIL-53(Cr)-NO_2 material (brown), exposed to SO_2 (red), and exposed to CO_2 (melon).

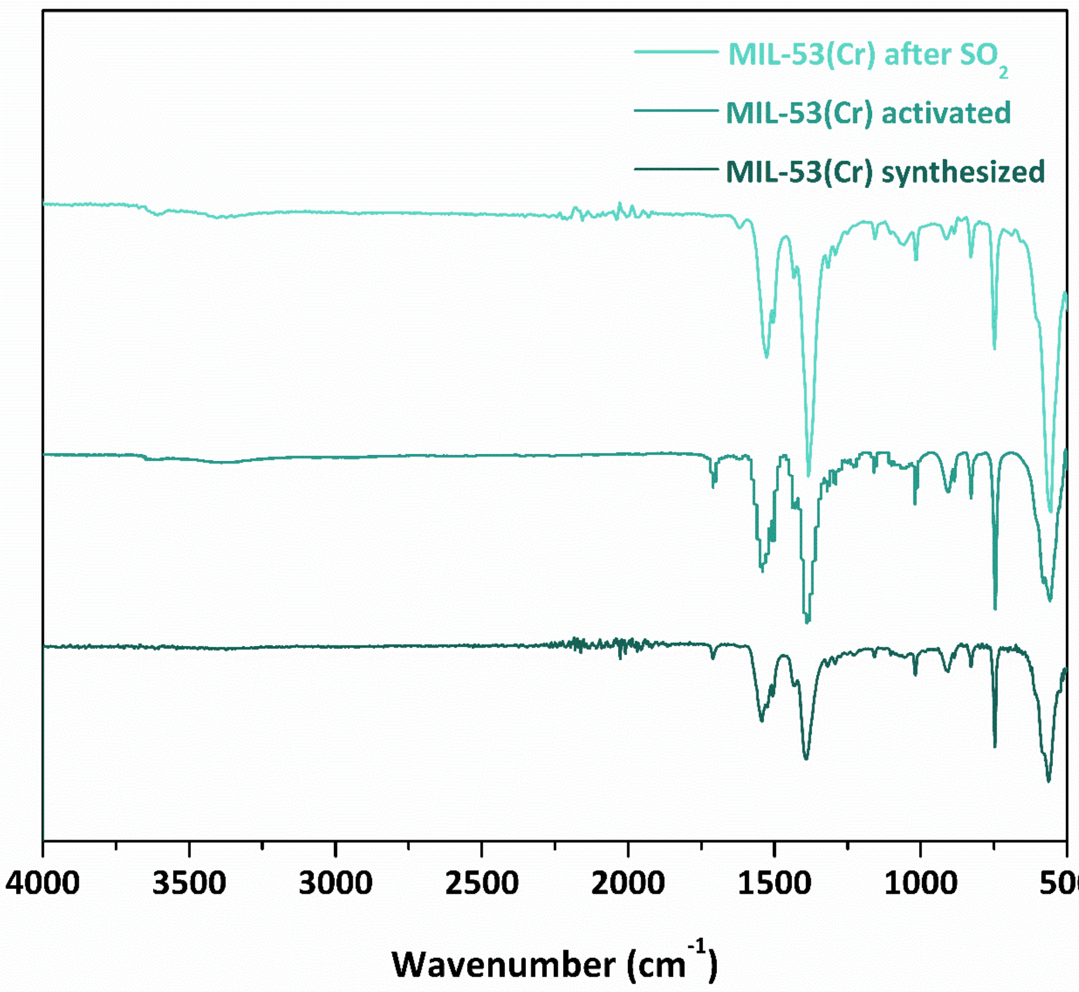

Figure S28. Comparison of the IR spectra of synthesized and washed (bright blue), activated (aqua), and SO_2 saturated (blue) MIL-53(Cr) samples.

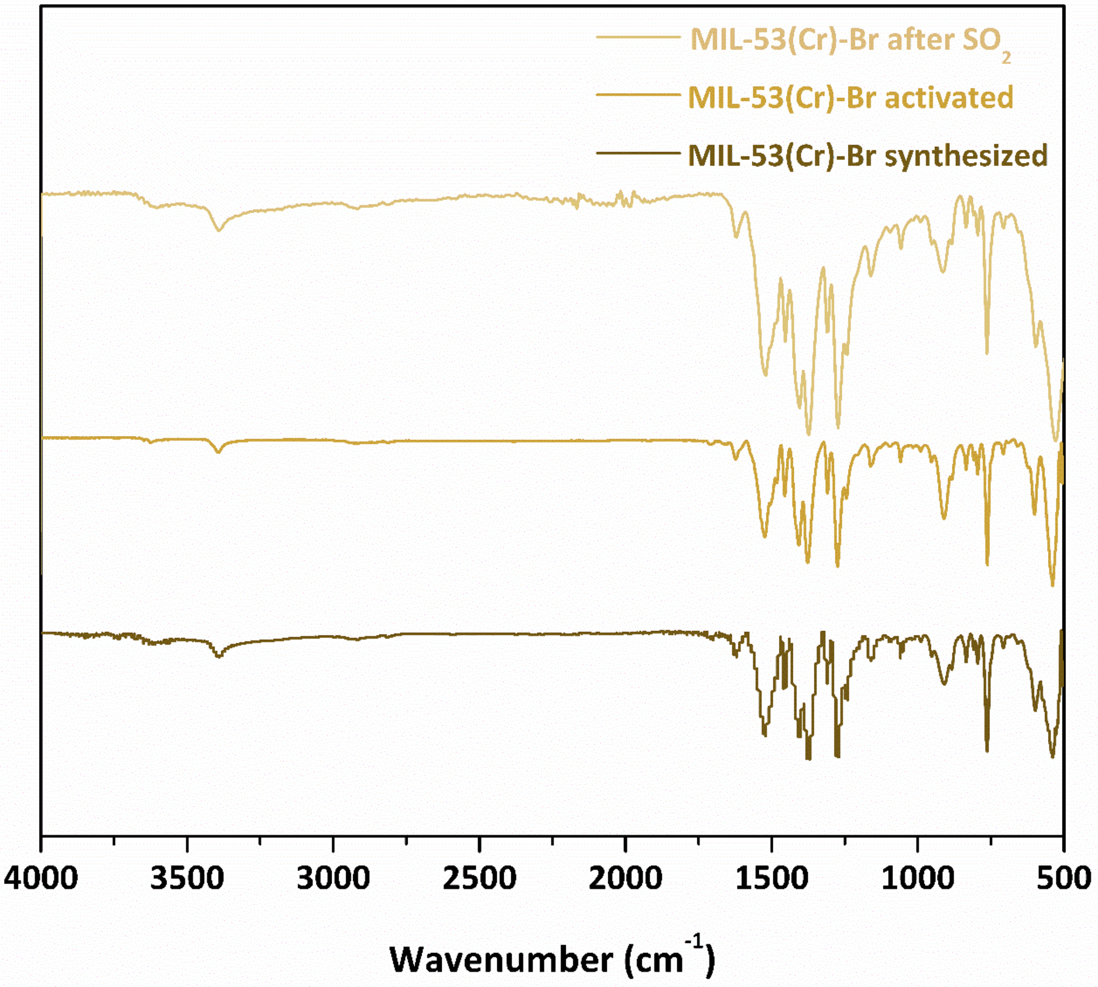

Figure S29. Comparison of the IR spectra of synthesized and washed (brown), activated (mustard), and SO_2 saturated (yellow) MIL-53(Cr)-Br samples.

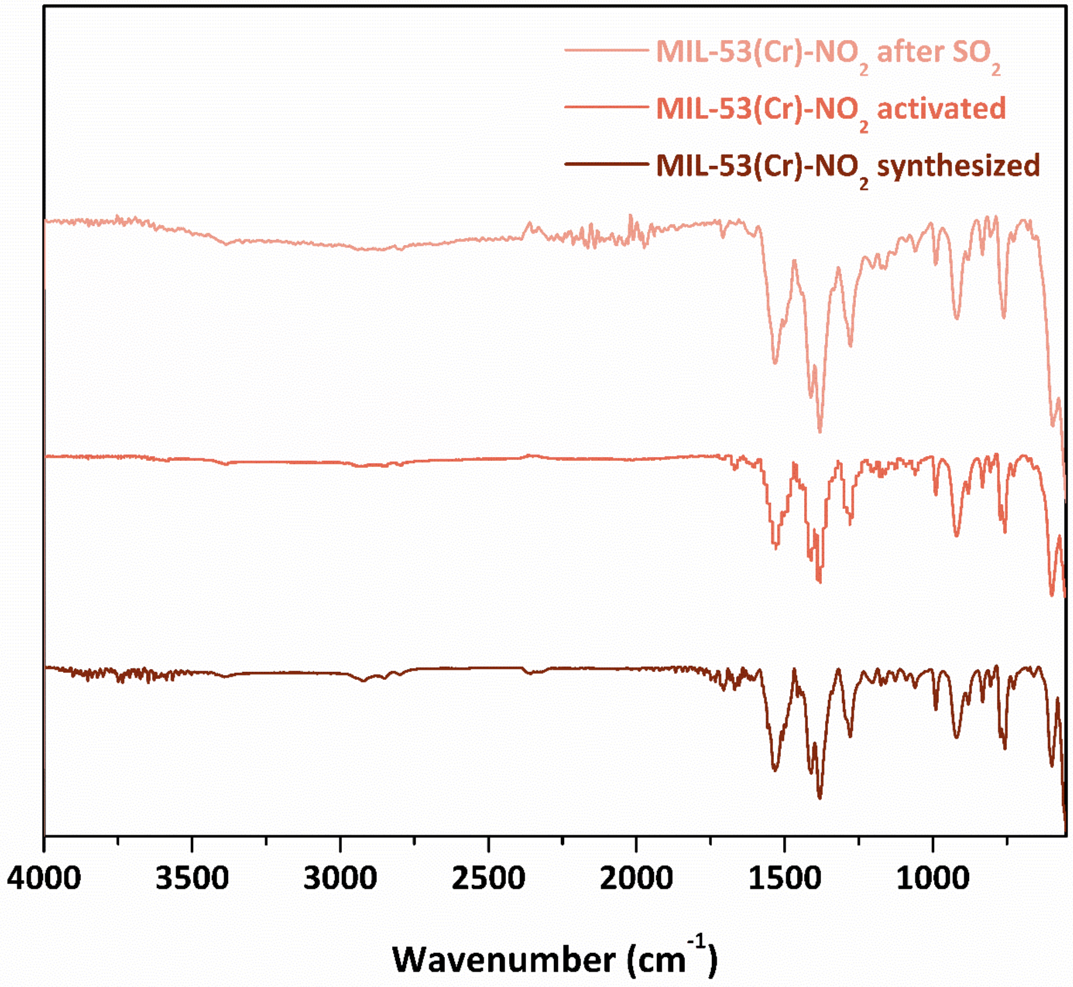

Figure S30. Comparison of the IR spectra of synthesized and washed (brown), activated (red), and SO_2 saturated (melon) MIL-53(Cr)-NO_2 samples.

S8. Comparison of SO_2 and CO_2 Isotherms

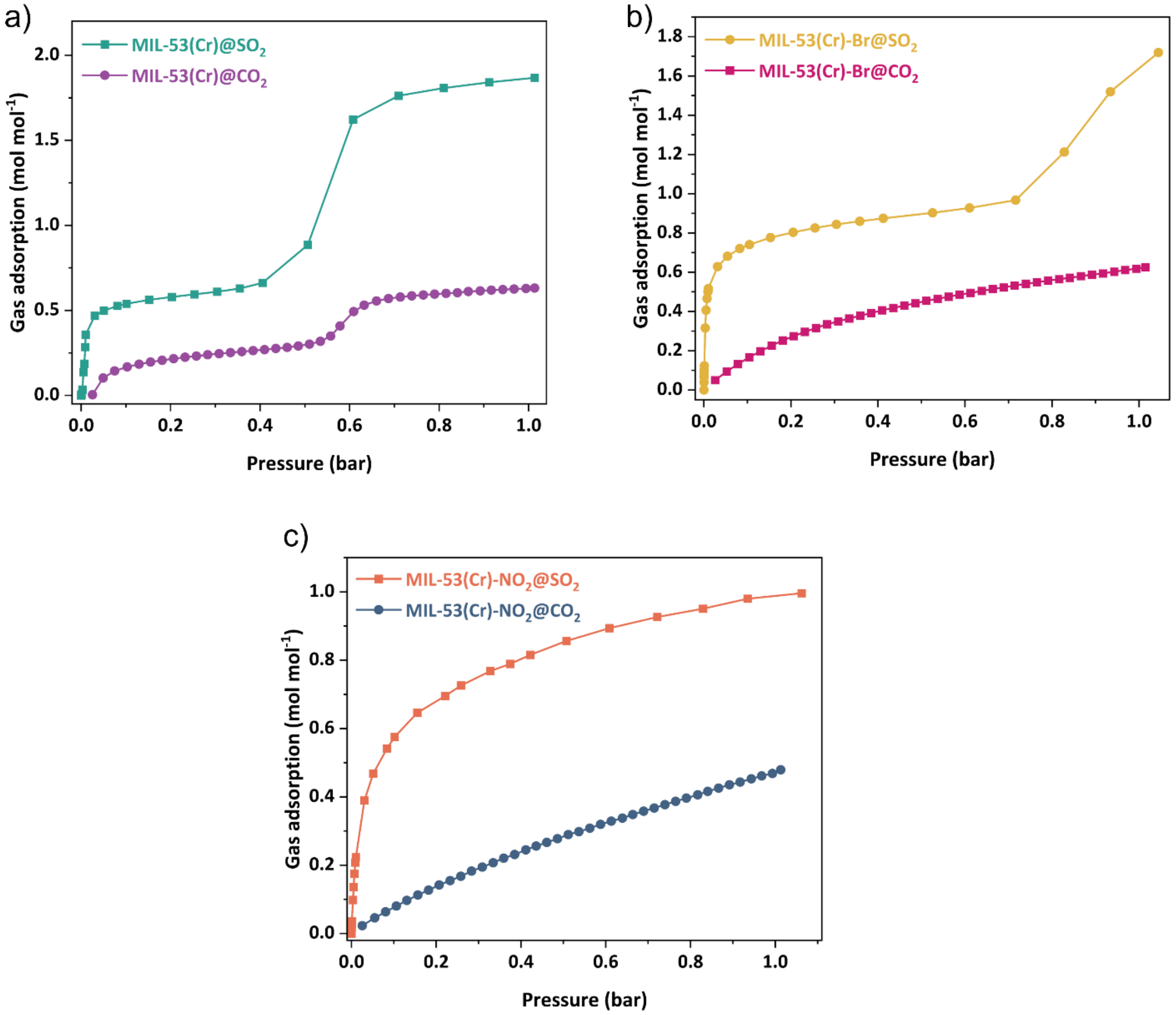

Figure S31. Comparison of SO_2 and CO_2 adsorption isotherms for a) MIL-53(Cr), b) MIL-53(Cr)-Br, and c) MIL-53(Cr)-NO_2. Circle points = SO_2 adsorption, square points = CO_2 adsorption. Isotherms were collected at 298 K.

S9. Ideal Adsorbed Solution Theory (IAST)

Predictions of the co-adsorption of SO_2:CO_2 mixtures on MIL-53(Cr), MIL-53(Cr)-Br, and NIL-53(Cr)-NO_2 were performed assuming the Ideal Adsorbed Solution Theory (IAST) assumptions as valid and using the Python package pyIAST.[S14]

The analytical Langmuir isotherm was fitted to the experimental CO_2 isotherms for all three materials and to the experimental SO_2 isotherm of MIL-53(Cr)-NO_2 with non-significant root mean square errors (Figure S32). None of the analytical models available in pyIAST fitted the experimental SO_2 isotherms of MIL-53(Cr) and MIL-53(Cr)-Br. Therefore, these SO_2 adsorption data were linearly interpolated, and the distributed pressures were calculated by numerical quadrature implemented in pyIAST (Figure S14). Therefore, the adsorption selectivity was calculated as:

$$S_{SO_2/CO_2} = \frac{x_{SO_2} y_{CO_2}}{x_{CO_2} y_{SO_2}}$$

where x_i and y_i are the mole fraction of components i = SO_2 and CO_2 in adsorbed and gas phase, respectively.

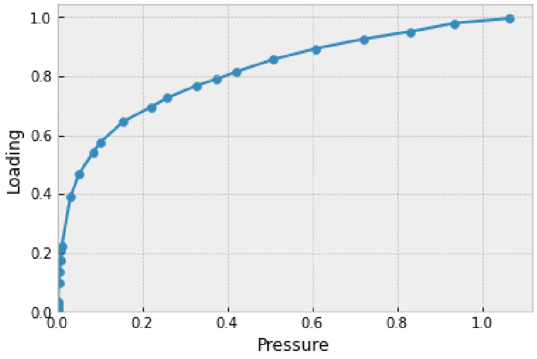

Figure S32. Experimental SO_2 adsorption isotherm of MIL-53(Cr)-NO_2 at 298 K (full circles) and Langmuir analytical isotherm fitted to experimental data (continuous line). Loading in mol/mol and Pressure in bar.

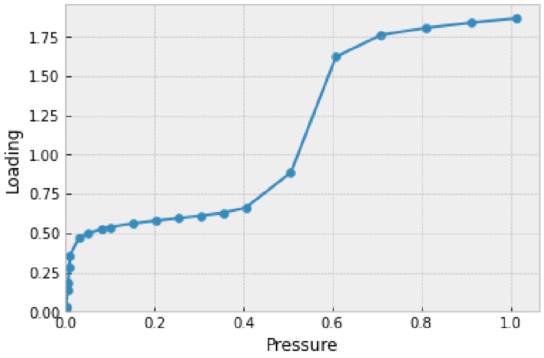

Figure S33. Experimental SO_2 adsorption isotherm of MIL-53(Cr) at 298 K (full circles) and the interpolation model fit (continuous line). Loading in mol/mol and Pressure in bar.

The selectivities of SO_2 versus CO_2 of different SO_2:CO_2 mole fractions at 1 bar pressure were calculated. The results are shown in Table S2.

Table S2. SO_2/CO_2 selectivities

SO_2/CO_2 composition	MIL-53(Cr)	MIL-53(Cr)-Br	MIL-53(Cr)-NO_2
0.1/99.90	57.34	14.33	29.67
0.5/99.50	10.4	2.95	6.13
1/99.00	6.68	1.52	3.14
5/95.00	1.56	0.33	0.71

S10. UV-Vis Spectroscopy

Before doing fluorescence experiments, solid UV experiments were performed on the three samples: MIL-53(Cr), MIL-53(Cr)-Br, and MIL-53(Cr)-NO_2, to know their possible excitation wavelengths, which were 295.5, 440, and 430 nm, respectively.

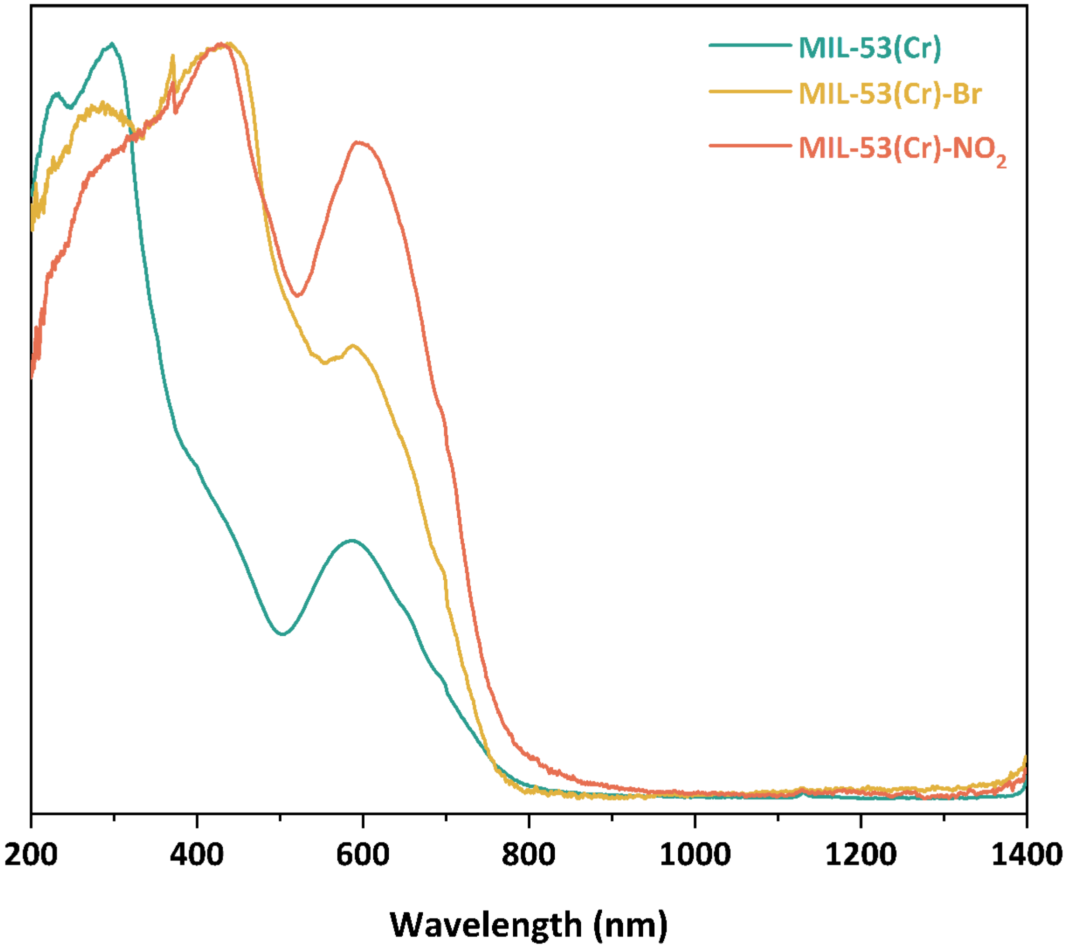

Figure S34. UV spectra of the activated samples of MIL-53(Cr) (blue), MIL-53(Cr)-Br (mustard) and MIL-53(Cr)-NO_2 (melon).

The excitation wavelengths were set to 300, 360, and 350 nm for MIL-53(Cr), MIL-53(Cr)-Br, and MIL-53(Cr)-NO_2, respectively, because these were the wavelengths at which the emission maximum was observed.

S11. Fluorescence Spectroscopy

After SO_2 saturation in our homemade saturation system, we moved the samples out of the system and immediately packed them into solid quartz holders which were inserted inside an Edinburgh FS5 Spectrofluorometer coupled to an SC-10 solid-state sample holder.

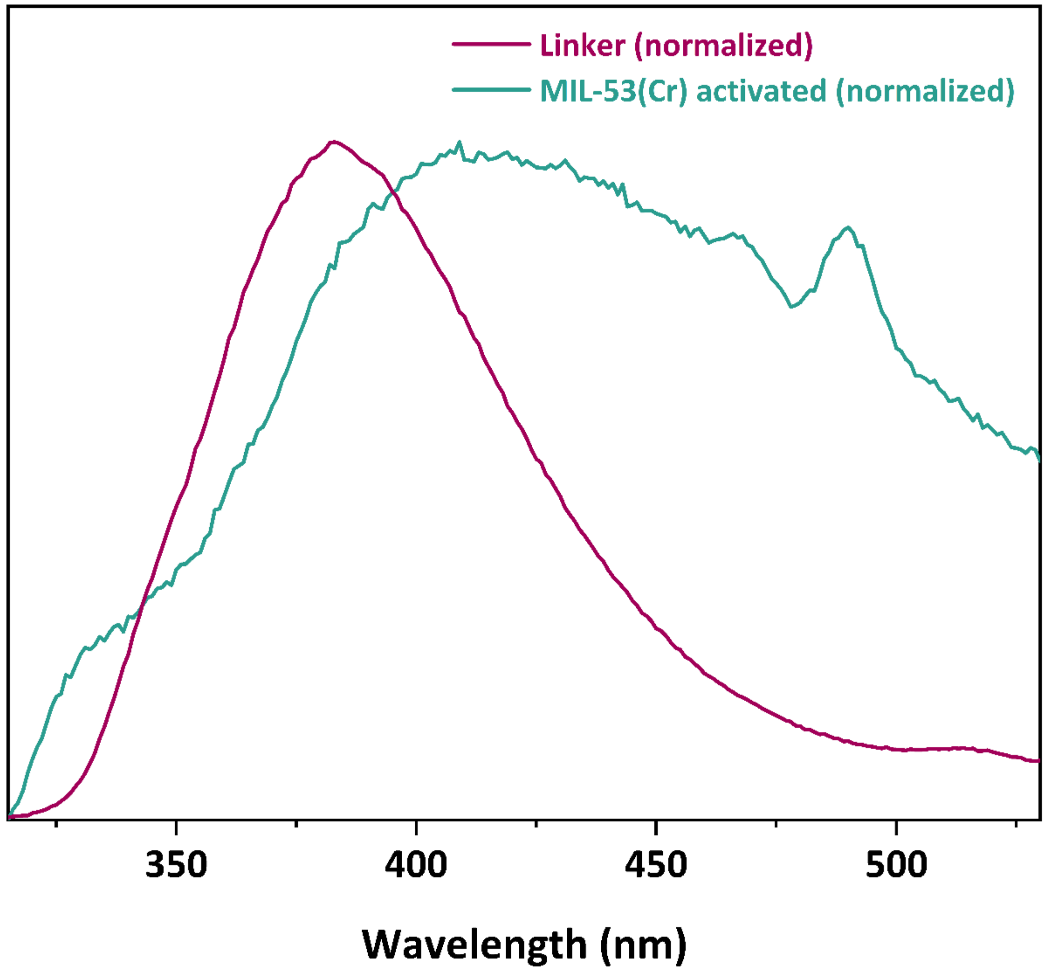

Figure S35. Comparison of the fluorescence spectra of BDC linker (purple) and MIL-53(Cr) material (blue).

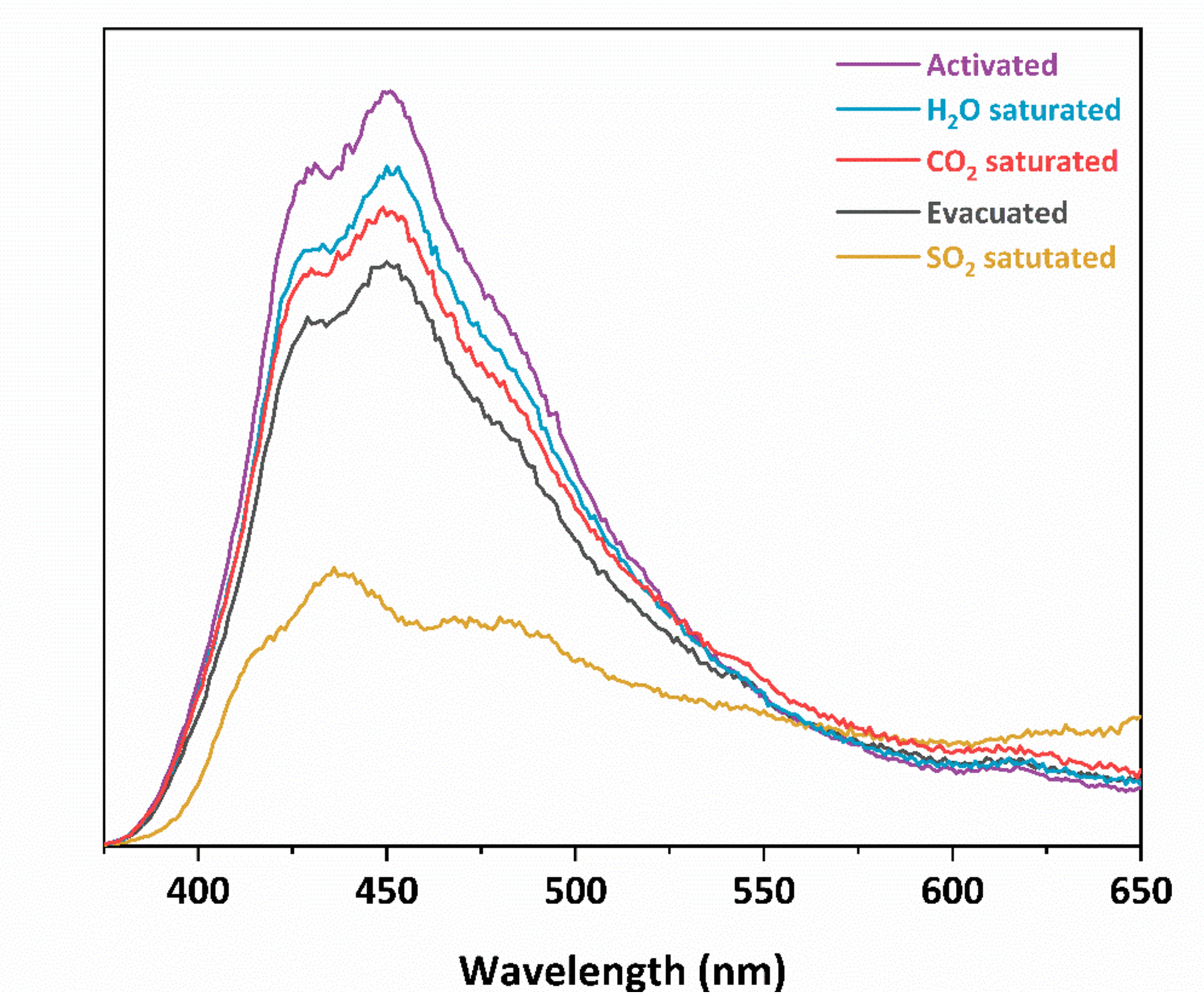

Figure S36. Comparison of the fluorescence spectra of activated (purple, H_2O saturated (blue), CO_2 saturated (red), washed and evacuated (gray), and SO_2 saturated (yellow) MIL-53(Cr)-Br samples.

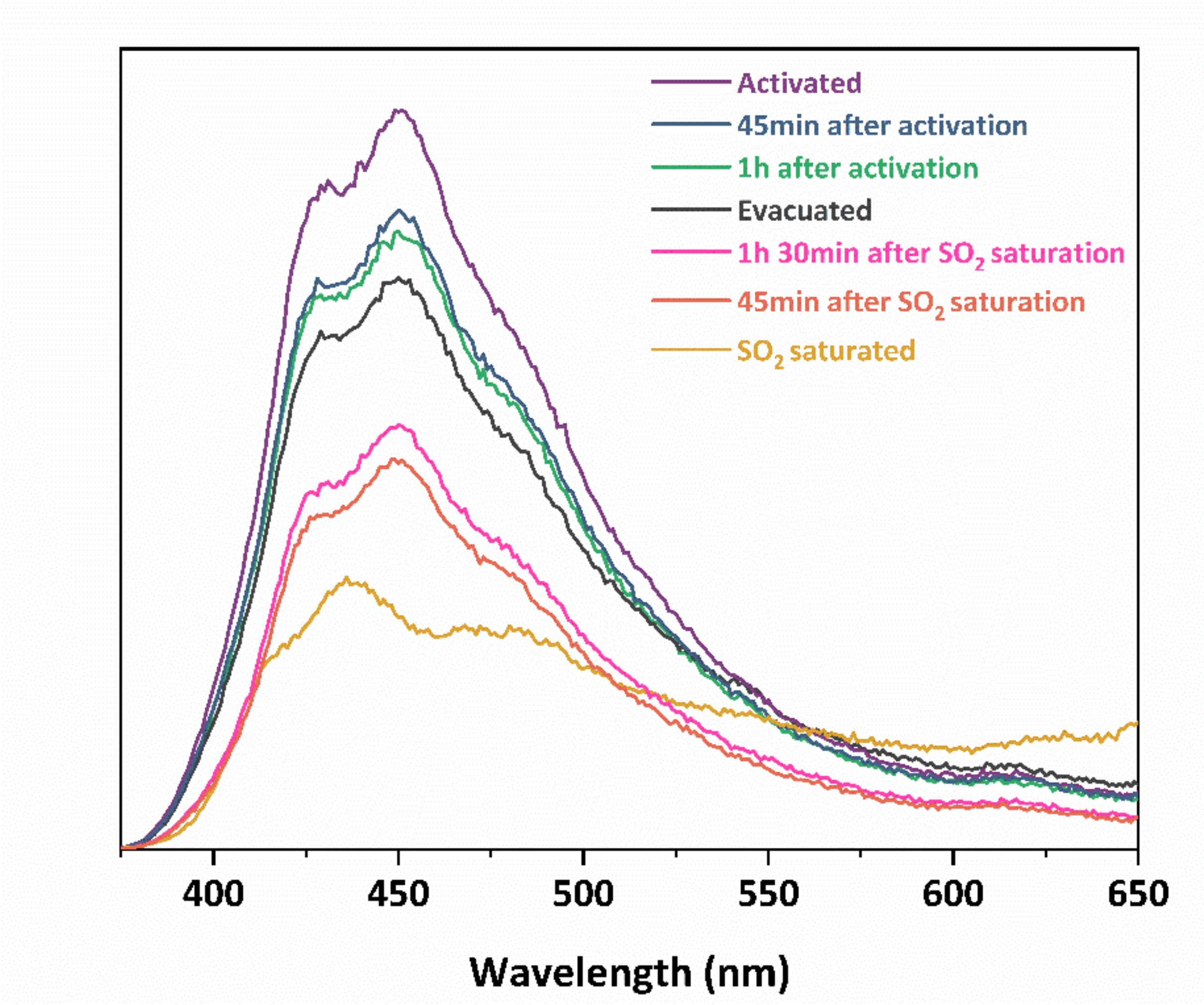

Figure S37. Comparison of the fluorescence spectra of activated (purple), 45 minutes after activation (blue), 1 hour after activation (green), washed and evacuated (gray), SO_2 saturated (yellow), 45 minutes after SO_2 saturation (orange), and 1 hour and 30 minutes after SO_2 saturation (pink) MIL-53(Cr)-Br samples.

It can be noted that, after a time following activation and SO_2 saturation, the fluorescence signal of the activated and saturated MIL-53(Cr)-Br samples approaches the signal of the washed and evacuated MIL-53(Cr)-Br sample. The same was observed for MIL-53(Cr) and MIL-53(Cr)-NO_2.

S12. References

S1. B. H. Toby and R. B. Von Dreele, *J. Appl. Crystallogr.* 2013, **46**, 544–549.

S2. J. W. M. Osterrieth, J. Rampersad, D. Madden, N. Rampal, L. Skoric, B. Connolly, M. D. Allendorf, V. Stavila, J. L. Snider, R. Ameloot, J. Marreiros, C. Ania, D. Azevedo, E. Vilarrasa-Garcia, B. F. Santos, X. -H. Bu, X. Zang, H. Bunzen, N. R. Champness, S. L. Griffin, B. Chen, R. -B. Lin, B. Coasne, S. Cohen, J. C. Moreton, Y. J. Colon, L. Chen, R. Clowes, F. -X. Coudert, Y. Cui, B. Hou, D. M. D'Alessandro, P. W. Doheny, M. Dincă, C. Sun, C. Doonan, M. T. Huxley, J. D. Evans, P. Falcaro, R. Ricco, O. Farha, K. B. Idrees, T. Islamoglu, P. Feng, H. Yang, R. S. Forgan, D. Bara, S. Furukawa, E. Sanchez, J. Gascon, S. Telalovic, S. K. Ghosh, S. Mukherjee, M. R. Hill, M. Munir Sadiq, P. Horcajada, P. Salcedo-Abraira, K. Kaneko, R. Kukobat, J, Kenvin, S. Keskin, S. Kitagawa, K. Otake, R. P. Lively, S. J. A. DeWitt, P. Llewellyn, B. V. Lotsch, S. T. Emmerling, A. M. Pütz, C. Martí-Gastaldo, N. Padial, J. García-Martínez, N. Linares, D. Maspoch, J. A. Suárez del Pino, P. Moghadam, R. Oktavian, R. E. Morris, P. S. Wheatley, J. Navarro, C. Petit, D. Danaci, M. J. Rosseinsky, A. P. Katsoulidis, M. Schröder, X. Han, S. Yang, C. Serre, G. Mouchaham, D. S. Shol, R. Thyagarajan, D. Siderius, R. Q. Snurr, R. B. Goncalves, V. Ting, J. L. Rowlandson, T. Uemura, T. Iiyuka, M. A. van der Veen, D. Rega, V. Van Speybroeck, S. M. J. Rogge, A. Lamaire, K. S. Walton, L. W. Bingel, S. Wuttke, J. Andreo, O. Yaghi, B. Zhang, C. T. Yavuz, T. S. Nguyen, Fe. Zamora, C. Montoro, H. Zhou, A. Kirchon and D. Fairen-Jimenez, *Adv. Mater.* 2022, **34**, 2201502.

S2. S. Bourelly, B. Moulin, A. Rivera, G. Maurin, S. Devautour-Vinot, C. Serre, T. Devic, P. Horcajada, A. Vimont, G. Clet, M. Daturi, J.- C. Lavalley, S. Loera-Serna, R. Denoyel, P. L. Llewellyn and G. Férey, *J. Am. Chem. Soc.*, 2010, **132**, 9488–9498.

S3. a) F. Millange, C. Serre and G. Férey, *Chem. Commun.*, 2002, **2**, 822–823; b) C. Serre, F. Millange, C. Thouvenot, M. Noguès, G. Marsolier, D. Louër, G. Férey, *J. Am. Chem. Soc.*, 2002, **124**, 13519–13526.

S4. L. Wu, G. Chaplais, M. Xue, S. Qiu, J. Patarin, A. Simon-Masseron and H. Chen, *RSC Adv.*, 2019, **9**, 1918–1928.

S5. D. Bara, E. G. Meekel, I. Pakamorė, C. Wilson, S. Ling and R. S. Forgan, *Mater. Horiz.* 2021, **8**, 3377–3386.

S6. A. Nuhnena and C. Janiak, *Dalton Trans.*, 2020, **49**, 10295–10307.

S7. S. Biswas, T. Ahnfeldt and N. Stock, *Inorg. Chem.*, 2011, **50**, 9518–9526.

S8. a) A. S. Munn, R. S. Pillai, S. Biswas, N. Stock, G. Maurin and R. I. Walton, *Dalton Trans.*, 2016, **45**, 4162–4168; b) T. Devic, P. Horcajada, C. Serre, F. Salles, G. Maurin, B. Moulin, D. Heurtaux, G. Clet, A. Vimont, J. M. Grenéche, B. le Ouay, F. Moreau, E. Magnier, Y. Filinchuk, J. Marrot, J. C. Lavalley, M. Daturi and G. Férey, *J. Am. Chem. Soc.*, 2010, **132**, 3, 1127–1136.

S9. A. López-Olvera, J. A. Zárate, E. Martínez-Ahumada, D. Fan, M. L. Díaz-Ramírez, P. A. Sáenz-Cavazos, V. Martis, D. R. Williams, E. Sánchez-González, G. Maurin and I. A. Ibarra, *ACS Appl Mater Interfaces*, 2021, **13**, 39363–39370.

S10. M. R. Tchalala, P. M. Bhatt, K. N. Chappanda, S. R. Tavares, K. Adil, Y. Belmabkhout, A. Shkurenko, A. Cadiau, N. Heymans, G. de Weireld, G. Maurin, K. N. Salama and M. Eddaoudi, *Nat. Commun.*, 2019, **10**, 1–10.

S11. S. S. Iremonger, R. Vaidhyanathan, R. K. Mah and G. K. H. Shimizu, *Inorg. Chem.*, 2013, **52**, 4124–4126.

S12. A. Schneemann, V. Bon, I. Schwedler, I. Senkovska, S. Kaskel and R. A. Fischer, *Chem. Soc. Rev.*, 2014, **43**, 6062–6096.

S13. C. M. Simon, B. Smit and M. Haranczyk, *Comput. Phys. Commun.*, 2016, **200**, 364.
